# The good, the bad, and the cringy: Introducing the EmpaToM-pos and EmpaToM-embarrass as tools for assessing empathy and theory of mind in diverse emotional contexts

**DOI:** 10.3758/s13428-026-03202-x

**Published:** 2026-11-02

**Authors:** Eva Landmann, Anne Böckler

**Affiliations:** Department of Psychology, Julius-Maximilians-Universität Würzburg, Röntgenring 11, 97070 Würzburg, Germany

**Keywords:** Social cognition, Social understanding, Theory of mind, Mentalizing, Empathy, Emotional context, Positive empathy, Empathic embarrassment

## Abstract

Empathizing and mentalizing are crucial to social understanding, yet few instruments capture these processes across a broad emotional spectrum. The EmpaToM is a video-based paradigm that independently measures empathy and theory of mind (ToM) and can additionally incorporate a prosociality measure. As its current form is limited to negatively valenced emotional contexts, we introduce two expanded German versions: EmpaToM-pos, featuring positively valenced narrations, and EmpaToM-embarrass, featuring embarrassing narrations, alongside the original neutral and negative conditions. In two pilot studies (*N*s = 28/27), we tested novel video stimuli and corresponding questions, revised them based on the data, and subsequently evaluated them in two higher-powered studies (*N*s = 60/55). Results confirmed successful empathy induction in all emotional conditions: Participants reported feeling worse after negative, more joyful after positive, and more embarrassed after embarrassing narrations (compared to all other valence conditions, respectively). Emotional valence also influenced prosociality: Negative narrations prompted the highest willingness to invest, but positive and embarrassing narrations also elicited greater prosociality than the neutral condition. Both expanded versions supported reliable ToM assessment without ceiling effects and with similar performance levels in the ToM and control conditions. Analyses further revealed that, on an interindividual level, empathic resonance in both negative and positive contexts was positively correlated with the degree of prosociality, highlighting the interrelation between empathizing and social decision-making. By incorporating positive and embarrassing emotional contexts, the EmpaToM-pos and EmpaToM-embarrass broaden the range of assessable social-emotional experiences, offering new opportunities to investigate empathy, ToM, and prosociality in more ecologically valid and diverse settings.

**Supplementary Information:**

The online version contains supplementary material available at https://doi.org/10.3758/s13428-026-03202-x.

## Introduction

Listening to a friend describe their latest amusement park adventure can feel a bit like being on an emotional rollercoaster oneself. Even if the listener never set foot in the park, they may still vicariously feel the narrator’s grave disappointment (when discovering their favorite ride is closed), delight (over the best ice cream of their life), and embarrassment (after vomiting that ice cream on the drop tower). These subjective experiences of sharing another person’s emotions align with a widely used definition that conceptualizes empathy as “an effective response that stems from the apprehension or comprehension of another’s emotional state or condition, and which is identical or very similar to what the other person is feeling or would be expected to feel” (Eisenberg et al., 2006, p. 647; see also De Vignemont & Singer, 2006). In simpler terms: Whatever our interaction partner feels, we feel it *with* them. Beyond empathy as an affective component, social understanding involves a conglomerate of cognitive processes often referred to as theory of mind (ToM), mentalizing, or cognitive perspective-taking (e.g., Frith & Frith, 2005, 2006; Ho et al., 2022; Schurz et al., 2014). ToM entails simulating or adopting another person’s perspective and inferring their thoughts, beliefs, action plans, and emotions, even when these are not explicitly expressed. Some studies indicate that both components of social understanding – feeling with others (empathy) and reasoning about others (ToM) – can enhance the subsequent willingness and ability to help or act kindly towards them (Ho et al., 2022; Lehmann et al., 2022; for a review, see Weisz & Cikara, 2021). In your friend’s case, feeling their emotions and understanding their goals may support gifting them a ticket to a park that finally gives them access to their favorite ride.

While empathy and ToM are not tied to any particular emotional context in theory, research (especially on empathy) has largely focused on negative emotions and experiences like pain and sadness (see Batson et al., 1987; Cuff et al., 2016; Morelli et al., 2015; Singer & Lamm, 2009). This bias is reflected and reinforced by commonly used experimental paradigms which tend to center on negative affect, ultimately limiting our comprehension of social understanding across the full spectrum of emotional experience. To help researchers further address this gap, we introduce an extended version of the EmpaToM paradigm (Kanske et al., 2015), a validated task that allows us to simultaneously measure empathy and theory of mind performance both on a behavioral and neural level in adolescents, healthy adults, and clinical populations (Breil et al., 2021; Lantos et al., 2023; Salazar Kämpf et al., 2022; Tholen et al., 2020). The extended tool broadens the emotional range to include not only neutral and negative contexts (e.g., ordinary work experiences or experiences of loss, respectively), but also positive and embarrassing contexts (e.g., joyful encounters or awkward public incidents, respectively). Next, we briefly outline prior research on social understanding in positive and embarrassing emotional settings and note what the expanded EmpaToM task adds.

### Social understanding in positive emotional contexts

While research on empathy has traditionally focused on negative emotions, feeling with others’ positive experiences (subsequently referred to as ‘positive empathy’, in contrast to ‘negative empathy’) seems equally, if not more, prevalent in real-world social interactions: In an experience sampling study that followed a representative US sample for 7 days, Depow and colleagues found that the majority of opportunities for empathy that participants encountered were positive (Depow et al., 2021). We may not frequently engage in tearful heart-to-hearts, but we regularly connect over shared joys: celebrating a friend’s achievements or congratulating our neighbors on becoming new parents. By now, several empathy measures have been developed to fill this gap, ranging from self-report questionnaires that inquire about the tendency to share other people’s positive as well as negative emotions (e.g., Perth Empathy Scale; Brett et al., 2023) to behavioral paradigms involving positively and negatively valenced emotional images (e.g., Multifaceted Empathy Test; Dziobek et al., 2008) or videos (e.g., a modified version of the Empathic Accuracy Task; Zaki et al., 2008).

In the investigation of ToM performance, classic tasks often employ stimulus material that is devoid of strong or task-relevant emotions (e.g., false-belief tasks, Wimmer & Perner, 1983; strange story tasks, Happé, 1994; the Imposing Memory Task, Kinderman et al., 1998; or tasks involving picture- or story-based action predictions, Brunet et al., 2000). However, some paradigms, while not explicitly manipulating valence, do involve more severe and task-relevant positive and negative emotions, for instance, when participants need to recognize (complex) mental states from face pictures (Reading the Mind in the Eyes Task, Baron‐Cohen et al., 2001), or infer actors’ motives in videotaped interactions (Movie for the Assessment of Social Cognition, Dziobek et al., 2008).

Like its negative counterpart, making sense of others’ positive experiences involves recognizing and sharing their emotions and understanding their perspective. It is therefore reasonable to assume that the affective and cognitive processes (i.e., empathy and ToM, respectively) when understanding others should be at least partially similar for negative and positive experiences. Indeed, some studies suggest that empathy with others’ negative and positive experiences relies on overlapping cognitive mechanisms and neural circuits (Andreychik & Migliaccio, 2015; Morelli et al., 2014), and both have been shown to facilitate prosocial behavior (Andreychik & Migliaccio, 2015). However, it may be an oversimplification to consider positive and negative empathy as simply two sides of the same coin. Rather than representing points on a single continuum, research suggests that positive empathy is distinct from negative empathy not only in specifics of neural correlates (Morelli et al., 2014), but also in developmental trajectories (Ziaei et al., 2021), personal dispositions (Andreychik & Migliaccio, 2015), and the kinds of helping behaviors it elicits (Andreychik & Lewis, 2017). These findings on positive empathy emphasize that a comprehensive understanding of empathy (and, likely, also ToM) requires the inclusion of diverse emotional contexts, an endeavor that may also provide valuable opportunities to deepen our understanding of the selective impairments of positive empathy and ToM observed in mental disorders (Morrison et al., 2016; Ohse et al., 2024) as well as ways to improve relationship quality (Andreychik, 2019) and promote prosocial behavior (Telle & Pfister, 2016).

### Social understanding in embarrassing emotional contexts

Empathic cringe is not only a familiar experience to all “funny fails compilation” enthusiasts, but has also been the subject of previous research which has underscored the existence of empathic embarrassment (i.e., feeling embarrassed *with* another person), and has examined factors that enhance or diminish this response, such as interpersonal liking, adopted perspective, and individual disposition (e.g., Marcus et al., 1996; Miller, 1987; Stocks et al., 2011). Overall, however, research on empathy and (even more so) ToM in embarrassing contexts has been rather sparse, although embarrassment presents a particularly interesting case for social understanding. For one, experiencing embarrassment is one of the few emotional states that is not unambiguously positive or negative in valence, but straddles the line between pain and humor, between abashment and amusement (e.g., Weisfeld & Weisfeld, 2014), making its perception, mirroring, and interpretation potentially more complex. For instance, embarrassment may not only be ambiguous for the person experiencing it, but also for observers, who may find themselves torn between wincing and laughing when a student accidentally calls their professor “mom”. Furthermore, embarrassment has been distinguished from basic emotions as a distinctly social emotion that is almost exclusively triggered when others perceive and/or evaluate how we perform (minor) violations of social norms, etiquette, or customs (e.g., Edelmann, 2012; Keltner, 1996; Miller, 1995; Tangney et al., 1996). Mental and behavioral responses to another’s social mishap may hence vary: On the one hand, it could elicit negative reactions, as the observed agent is violating established standards and attracting negative attention. On the other hand, closeness and trust could be fostered because, after all, the person’s embarrassment signals their adherence to social norms (Keltner & Buswell, 1997; Tangney et al., 1996).

Again, the assumption that social understanding in embarrassing and in basic negative emotional contexts relies on (partly) overlapping processes and mechanisms seems reasonable. Although they differ in emotional valence and complexity, both settings entail meaningful social information (e.g., some degree of self-disclosure) and may be especially prone to eliciting emotional resonance and perspective taking. In fact, though most research on empathy in embarrassing contexts has developed separately from broader investigations in the field of empathy, some results suggest that empathy for embarrassment and for unambiguously negative emotions share similarities. For example, in their innovative studies, Krach and colleagues (2011) employed written vignettes displaying various situations in which an agent violated social etiquette. The authors found activation in brain regions typically reported for empathy for pain and sadness, including components of the so-called pain matrix (e.g., Krach et al., 2011). Interestingly, people feel vicarious embarrassment even for those who do not show signs of embarrassment themselves (e.g., because they are unaware of the situation or do not care). According to our and others’ definition, this does not constitute an empathic response in the narrower sense (e.g., Paulus et al., 2013; Wondra & Ellsworth, 2015), as the involved individuals’ emotions mismatch. While perspective taking takes place in either setting, emotion sharing only occurs when the observed person also feels the embarrassment – a difference that is reflected in the corresponding neural correlates (Mayer et al., 2020; Paulus et al., 2015).

To our knowledge, there are no published studies and available tasks that systematically compare empathy and/or ToM for embarrassing experiences to empathy and/or ToM for other emotions. In favor of a more comprehensive investigation of the mental processes, neural mechanisms, individual differences, and behavioral consequences (e.g., helping behavior) of empathy and ToM, we propose an extension of the established EmpaToM paradigm with positive and embarrassing contexts.

### Expanding the EmpaToM

The EmpaToM (Kanske et al., 2015, 2016) is an established tool designed to assess the complex processes of social affect and cognition by simultaneously measuring empathy and theory of mind^1^. Participants see naturalistic video stimuli in which individuals recount short autobiographical experiences, followed by questions targeting the participant’s affective state and comprehension of the narration. The stories either recount neutral events (e.g., hobbies, work, and daily routines) or emotionally negative experiences (e.g., losing one’s job, illness of a relative), serving as a manipulation of emotional valence. If participants report more negative affect after negative compared to neutral videos, this is interpreted as an empathic response. ToM performance is assessed with questions that require participants to adopt the speaker’s perspective and infer their thoughts, beliefs, or intentions. These are contrasted with control questions that focus on factual reasoning about the story but do not require mentalizing. The two experimental factors – emotional valence (neutral vs. negative) and ToM requirement (ToM vs. no-ToM) – are fully crossed across trials, allowing for comparisons of their effects and interactions on both behavioral and neural levels.

While, to our knowledge, this is the first study to expand the EmpaToM’s emotional valence conditions, several successful adaptations of the original paradigm already exist. These include an English-language version (Lantos et al., 2023) and an adolescent-specific adaptation (Breil et al., 2021). Other studies have extended the paradigm to incorporate measures of prosocial behavior, an important correlate of both empathizing and mentalizing (e.g., Lehmann et al., 2022; McDonald & Kanske, 2023). The EmpaToM’s versatility is further demonstrated by its widespread use across diverse research areas over the past decade, including investigations of neural correlates (Kanske et al., 2016; Molenberghs et al., 2016; Tholen et al., 2020), stress-related processes (Blasberg et al., 2022; Schulreich et al., 2022), age-related differences (Pollerhoff et al., 2022), and traits associated with clinical conditions (Choi et al., 2025; Jauk et al., 2024). As an additional advantage, the multimodal and dynamic nature of the EmpaToM videos allows targeted manipulations of the provided visual and/or auditory information and the assessment of their relative impact on empathy and ToM (e.g., Landmann et al., 2024, 2025).

Given this proven flexibility and robustness, expanding the EmpaToM beyond negatively valenced emotional contexts marks an important step toward a more comprehensive assessment of empathy and mentalizing. Our extension of the EmpaToM with positively valenced (EmpaToM-pos) and embarrassing narrations (EmpaToM-embarrass) enables the simultaneous assessment and comparison of affective empathy and ToM across emotional contexts, using dynamic video stimuli that are both naturalistic and experimentally controlled. By including the measure of prosociality, we can additionally present initial evidence on how feeling with and mentalizing about another person relate to the willingness to act in their favor in emotionally distinct settings, and examine whether the expected association between (negative) empathic resonance and prosociality emerges, thereby offering further support for the paradigm’s validity.

## Transparency and openness

We report how we determined our sample size, all data exclusions (if any), all manipulations, and all measures in the study. The design and analysis of the study were preregistered (https://osf.io/zaumq). All data, and analysis scripts are openly available on PsychArchives (pilot data: https://doi.org/10.23668/psycharchives.22644; task validation data: https://doi.org/10.23668/psycharchives.22645; code: https://doi.org/10.23668/psycharchives.22646). The repository also contains the complete videos used in the final versions of the EmpaToM-pos and EmpaToM-embarrass tasks as presented in this paper (https://doi.org/10.23668/psycharchives.22648), as well as transcripts of all narration sets and corresponding mentalizing/factual reasoning questions (https://doi.org/10.23668/psycharchives.22647).

The procedure adhered to the ethical standards outlined in the 1964 Declaration of Helsinki concerning the treatment of human participants in research. The paradigm used in this study (the EmpaToM task) received ethical approval from the local ethics committee (Ethikkommission des Institutes für Psychologie der Humanwissenschaftlichen Fakultät der Julius-Maximilians-Universität Würzburg, GZEK 2023-36).

During the preparation of this manuscript, the authors used ChatGPT-4o (OpenAI, 2024) in order to improve readability and language quality. The authors reviewed and edited all content and take full responsibility for the final published version.

## Pilot study

### Method

#### Sample

A total of 61 participants took part in the pilot experiments, which were conducted online. Of these, 31 participants completed Pilot 1 (positive condition in addition to neutral and negative), and 30 completed Pilot 2 (embarrassing condition in addition to neutral and negative). Participants were recruited via the university’s recruitment platform or through private contacts and received course credit for their participation. In line with the preregistered criteria, we excluded six participants from the analyses: two because they reported knowing at least one of the narrators depicted in the videos, and four due to poor performance on the single-choice questions (i.e., accuracy more than 2 standard deviations below the experiment-specific mean). The final sample therefore consisted of 28 participants in Pilot 1 (*M*_age_ = 24.2 years, *SD*_age_ = 8.8; 82% female) and 27 in Pilot 2 (*M*_age_ = 24.2 years, *SD*_age_ = 8.5; 89% female, 4% diverse).

#### Study material

We selected eight sets of narrations from the original EmpaToM, ensuring a balanced representation of male and female narrators of diverse ages. Each original set contained four short autobiographical narrations in German told by one narrator, corresponding to all combinations of two dichotomous factors: emotional *valence* (neutral vs. negative) and *ToM requirement* (ToM vs. noToM). The neutral narrations focused on topics like daily routines or work life, while the negative ones described adverse experiences, e.g., job loss or health issues. For each valence category, one narration required mentalizing (e.g., inferring intentions or beliefs, ToM condition), while the other required factual reasoning (noToM condition).

To extend the EmpaToM, we added two additional valence conditions to each set: positive and embarrassing. As with the original conditions, we created one ToM and one noToM narration per new valence condition. The positive narrations included events such as professional success, happy family occasions, reunions with old friends, and athletic achievements. Embarrassing narrations described moments of social discomfort, including public clumsiness, awkward miscommunication, mistaking a stranger for someone familiar, or being humiliated in front of a romantic interest. We ensured internal coherence within each set, avoiding contradictions in narrative content or character behavior. Two transcribed example sets are provided in Supplement S1, while the final sets of all eight narrators (eight narrations each, available as videos) are accessible via PsychArchives (https://doi.org/10.23668/psycharchives.22648).

Amateur actors performed all narrations, each recording their assigned set with their face and upper body visible against a neutral white background. The clips were later cropped to a 4:3 format, ensuring consistent framing and comparable apparent height of the narrators across videos. Each video lasted between 12 and 15 seconds.

Each narration was followed by a comprehension question requiring either mentalizing (“[Name] thinks that…”) or factual reasoning (“It is true that…”). For the newly added valence conditions, we created questions that matched the style and structure of the original items. We also confirmed that the response options did not differ significantly across conditions in terms of linguistic features such as word count, use of the subjunctive mood, or passive constructions.

#### Procedure

The experiment was programmed using PsychoPy (Peirce et al., 2019) and conducted online via Pavlovia (pavlovia.org). Participants completed the study on a laptop or desktop computer and were randomly assigned to one of two versions of the experiment, each incorporating a different emotional context (positive or embarrassing, respectively) alongside the original ones (neutral and negative). In Pilot 1, participants viewed eight sets of narrations with neutral, negative, and positive emotional valence. In Pilot 2, the narrations included neutral, negative, and embarrassing content.

After providing informed consent and demographic information, participants received task instructions and completed two practice trials (one neutral and one negative) to familiarize themselves with the procedure and the emotional tone of the narrations. After these practice trials, participants could discontinue if they experienced technical difficulties or felt the emotional content might be too distressing. Those who chose to continue then completed 48 experimental trials presented in randomized order, which took approximately 40 min.

**Fig. 1:**
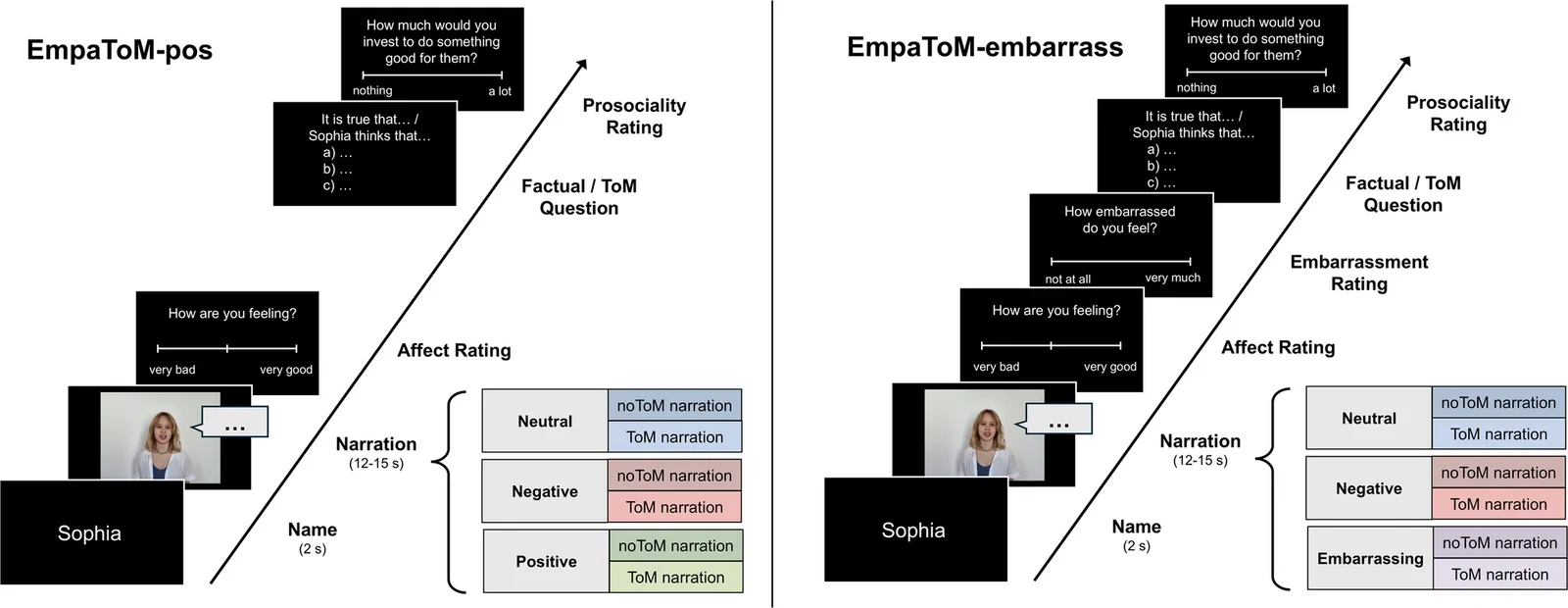
Schematic trial sequence. *Note.* Participants completed 48 trials in which they watched short autobiographical narrations and then answered questions. Narrations were either emotionally neutral, negative, positive (EmpaToM-pos: Experiment 1/Pilot 1), or embarrassing (EmpaToM-embarrass: Experiment 2/Pilot 2), and required either theory of mind (ToM) reasoning or factual reasoning to answer the question. We collected embarrassment ratings only in the EmpaToM-embarrass

Each trial (for a schematic depiction, see Fig. 1) began with the name of the narrator displayed for 2 s, followed by a short autobiographical video clip (12–15 s) of the narrator sharing a short autobiographical story in one of six conditions (ToM requirement: noToM vs. ToM × valence: neutral vs. negative vs. positive/embarrassing [Pilot 1/Pilot 2]). After watching the video, participants rated their current affective state (“How are you feeling?”; in German: “Wie fühlen Sie sich?”) on a scale from – 3 (“very bad”) to 3 (“very good”). In Pilot 2, they also indicated how embarrassed they felt (“Wie peinlich berührt sind Sie?”) on a scale from 0 (“not at all”) to 6 (“very much”).

Following the ratings, participants answered a single-choice question assessing their understanding of the narration. In the noToM condition, questions required factual reasoning and began with “It is true that…”, whereas in the ToM condition, they required mentalizing and began with “[Name] thinks that…”. Below the question stem, three possible continuations (a, b, c) were presented, from which participants were asked to select the one they found most fitting. Finally, participants indicated how many resources they would be willing to invest to do something good for the narrator (“How many of your resources would you invest to do something good for the person in the video?”, in German: “Wie viele Ihrer Ressourcen würden Sie investieren, um der Person im Video etwas Gutes zu tun?”) on a scale from 0 (“nothing”) to 9 (“a lot”). Note that, unlike in the original EmpaToM, responses were not time-limited, as our study prioritized unrestricted judgments and performance over assessment of precise temporal dynamics of responding, which are mainly relevant in fMRI or EEG studies.

After completing the 48 trials, participants had the opportunity to report any technical difficulties or note anything unusual in a free-text field. Finally, they were asked whether they recognized any of the narrators, thanked for their participation, and debriefed.

#### Design and analysis

As dependent variables, we assessed (i) affect rating – with the affect difference between neutral and emotional narrations being interpreted as empathic responding – (ii) accuracy in the single-choice questions requiring either ToM or factual reasoning, (iii) response time (RT) in trials in which these questions were correctly answered, and (iv) prosociality, i.e., the amount of hypothetical resources participants were willing to invest to do some good for the narrator. In Pilot 2, we additionally analyzed (v) the embarrassment participants reported. Trials with RTs exceeding 120 s were excluded from the analysis of RTs, as such durations were unlikely to reflect valid responses to the question.

Each experiment featured two within-subject factors: The first was the three-level factor *valence*, which included the two original EmpaToM conditions (neutral and negative) as well as one additional valence condition specific to each experiment (positive in Pilot 1, embarrassing in Pilot 2). Thus, Pilot 1 compared neutral, negative, and positive valence conditions, whereas Pilot 2 compared neutral, negative, and embarrassing ones. Fully crossed with valence was the two-level factor *ToM requirement*: In half of the trials, participants were required to engage in mentalizing (ToM condition), while in the other half, understanding the narration only required factual reasoning (noToM condition).

For each experiment, we conducted 3 (valence: neutral, negative, positive/embarrassing) × 2 (ToM requirement: noToM, ToM) repeated-measures analyses of variance (ANOVAs), using RStudio (R Core Team, 2024) with the packages ‘afex’ (Singmann et al., 2024), ‘psych’ (Revelle, 2024), and ‘rstatix’ (Kassambara, 2023). In cases where the assumption of sphericity was violated, we applied Greenhouse-Geisser corrections. Post-hoc comparisons were performed using Bonferroni-corrected *t* tests.

## Results

### Affect rating

**Fig. 2:**
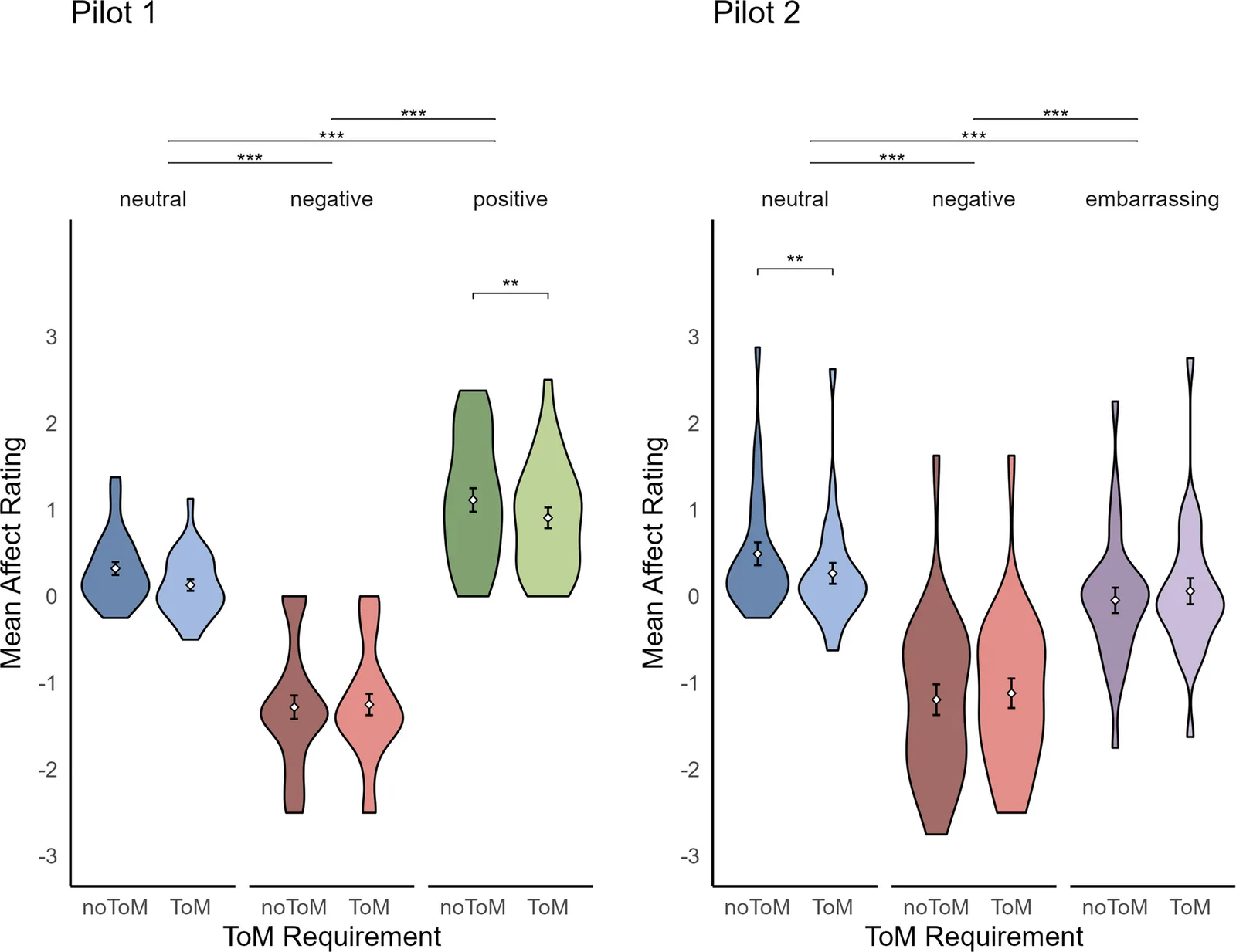
Affect ratings in Pilot 1 and Pilot 2. *Note*. Violin plots of mean affect ratings (on a scale from – 3 to + 3) in Pilot 1 (*left*) and Pilot 2 (*right*). *Diamonds* indicate the mean, and *error bars* represent standard errors. The *x*-axis depicts the different combinations of valence and ToM requirement. *Horizontal brackets* indicate significant differences between valence conditions (*upper brackets*) and between ToM conditions within each valence × ToM requirement combination (*lower brackets*). *p <.05, **p <.01, ***p <.001

Affect rating results are displayed in Fig. 2. In Pilot 1, affect ratings varied significantly across the three valence conditions (neutral, negative, and positive), *F*(1.1, 29.3) = 96.25, *p* < .001, ${\eta}_{p}^{2}$ = .78: As expected, participants reported significantly more negative affect following negative narrations compared to neutral ones, *t*(27) = 10.24, *p* < .001, *d* = 1.94, and significantly more positive affect following positive narrations relative to neutral ones, *t*(27) = – 7.96, *p* < .001, *d* = – 1.50, and negative ones, *t*(27) = – 9.94, *p* < .001, *d* = – 1.88. In Pilot 2, we again observed significant differences in affect ratings across valence conditions, *F*(1.4, 36.3) = 85.67, *p* < .001, ${\eta}_{p}^{2}$ = .77, replicating the pattern of lower affect after negative compared to neutral narrations, *t*(26) = 10.00, *p* < .001, *d = *1.92. Following embarrassing narrations, affect was lower than following neutral ones, *t*(26) = 4.60, *p* < .001, *d* = 0.88, but higher than after negative narrations, *t*(26) = – 9.54, *p* < .001, *d* = – 1.84.

In addition to the substantial valence effects, ToM requirement also had a small but significant influence on participants’ affect rating. In Pilot 1, this was reflected in a main effect, *F*(1, 27) = 8.05, *p* = .009, η^2^ₚ = .23, and an interaction, *F*(2, 54) = 4.57, *p* = .015, η^2^ₚ = .14, driven by the positive condition, in which noToM narrations elicited significantly higher affect than ToM narrations, *t*(27) = 3.31, *p* = .009, *d* = 0.63, whereas no significant differences emerged in the other valence conditions (*p*s >.096). In Pilot 2, the valence × ToM requirement interaction reached significance, *F*(2, 52) = 6.25, *p* = .004, η^2^ₚ = .19, while the main effect of ToM requirement was not significant, *F*(1, 26) = 0.16, *p* = .691, η^2^ₚ = .01. This interaction appeared to be driven by the neutral condition, in which noToM narrations elicited significantly more positive affect than ToM narrations, *t*(26) = 3.51, *p* = .006, *d* = 0.67 (a typical pattern, see Kanske et al., 2015); no differences were observed in the negative or embarrassing conditions (*p*s > .525).

In addition to these general analyses, we examined affect ratings individually for each narrator set (i.e., the eight videos provided by each speaker) and identified two sets, namely the set of the narrators ‘Tobias’ and ‘Klara’, that appeared less effective in eliciting positive empathy (for details, see Supplement S2). In these cases, either the positive narrations did not generate sufficiently positive affect, or the difference in affect ratings between the neutral and positive conditions was not clearly pronounced.

#### Embarrassment rating

**Fig. 3:**
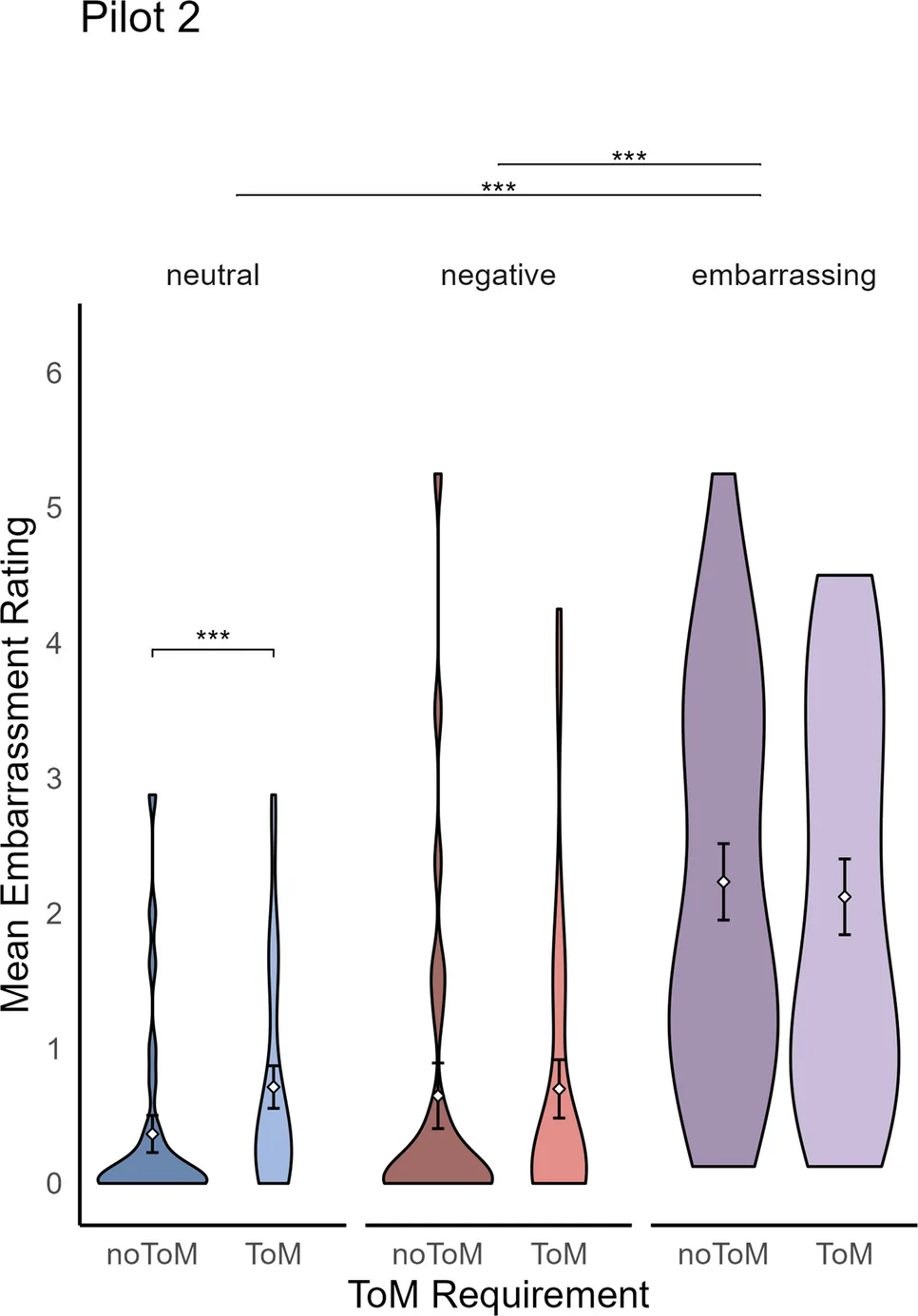
Embarrassment ratings in Pilot 2. *Note.* Violin plot of mean embarrassment ratings (on a scale from 0 to 6) in Pilot 2. *Diamonds* indicate the mean, and *error bars* represent standard errors. The *x*-axis depicts the different combinations of valence and ToM requirement. *Horizontal brackets* indicate significant differences between valence conditions (*upper brackets*) and between ToM conditions within each valence × ToM requirement combination (*lower brackets*). *p <.05, **p <.01, ***p <.001

In Pilot 2, we additionally assessed participants’ embarrassment (see Fig. 3). As expected, embarrassment ratings differed significantly across valence conditions, *F*(2, 52) = 35.83, *p* < .001, ${\eta}_{p}^{2}$ = .58. Participants reported feeling more embarrassed after embarrassing narrations compared to both neutral, *t*(26) = – 7.54, *p* < .001, *d* = – 1.54, and negative narrations, *t*(26) = – 6.44, *p* < .001, *d* = – 1.24. There was no significant difference between the neutral and negative conditions (*t* < 1). ToM requirement also slightly affected embarrassment ratings, as indicated by a significant main effect, *F*(1, 26) = 4.63, *p* = .041, ${\eta}_{p}^{2}$ = .15, and a valence × ToM requirement interaction, *F*(1.6, 41.3) = 8.47, *p* = .002, η^2^ₚ = .25. Specifically, in the neutral condition, participants reported greater embarrassment following ToM narrations compared to noToM narrations, *t*(26) = – 4.74, *p* < .001, *d* = – 0.91, whereas no such differences were observed in the negative or embarrassing conditions (all *p*s > .699).

#### Performance in single-choice task

While analyses of affect and embarrassment ratings were central to assessing whether empathy was successfully induced, we also examined participants’ performance on the single-choice questions, which required either mentalizing (ToM condition) or factual reasoning (noToM condition). The primary aim was to evaluate whether the newly developed questions were of appropriate difficulty (i.e., neither too easy nor too difficult) and to assess whether systematic differences between conditions existed that could be disadvantageous (e.g., if questions in negative trials were consistently more difficult than those in positive trials).

##### Accuracy

**Fig. 4:**
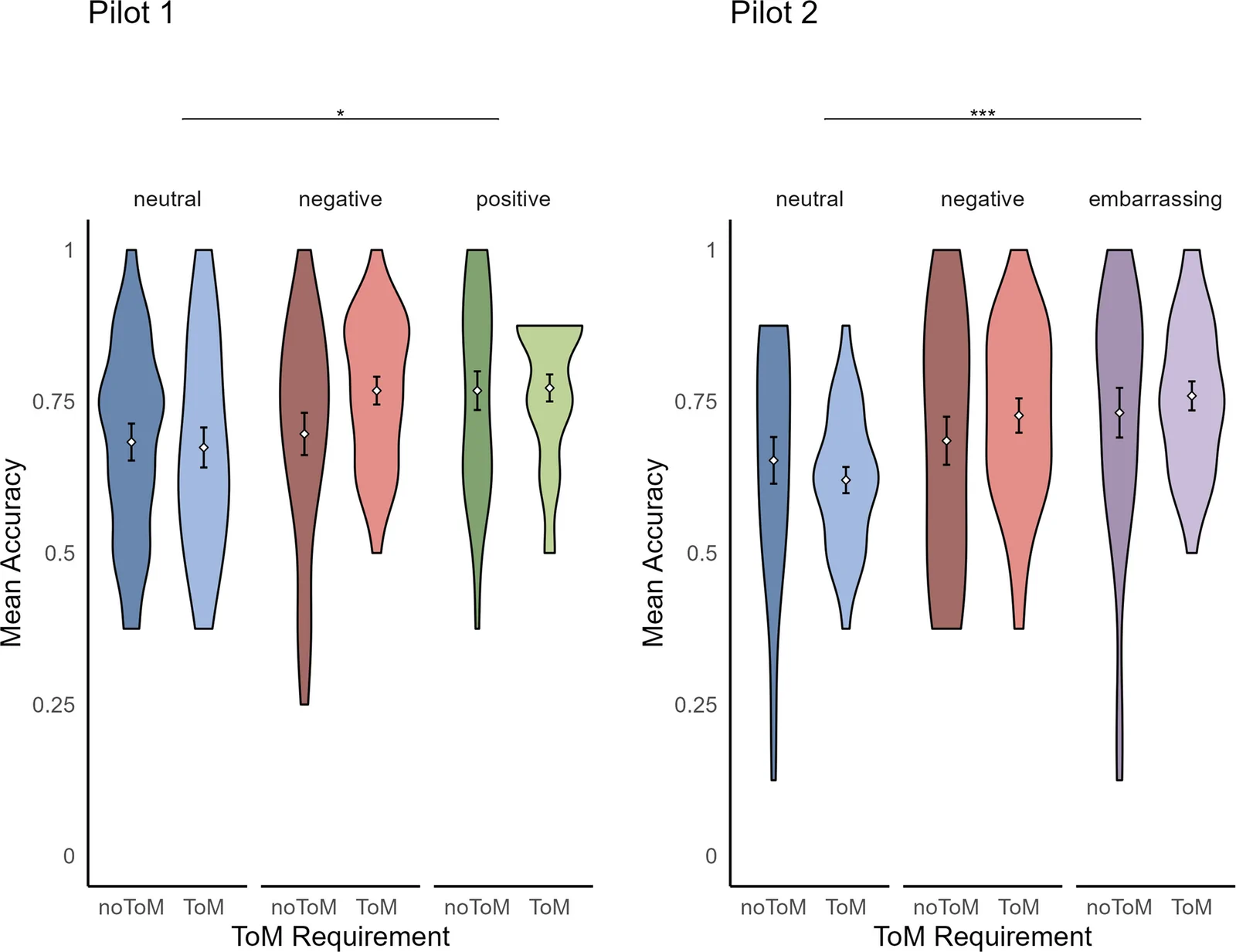
Accuracy in Pilot 1 and Pilot 2. *Note.* Violin plots of mean accuracy (from 0 to 1) in the single-choice questions in Pilot 1 (*left*) and Pilot 2 (*right*). *Diamonds* indicate the mean, and *error bars* represent standard errors. The *x*-axis depicts the different combinations of valence and ToM requirement. *Horizontal brackets* indicate significant differences between valence conditions (*upper brackets*) and between ToM conditions within each valence × ToM requirement combination (*lower brackets*). *p <.05, **p <.01, ***p <.001

Accuracy results are displayed in Fig. 4. In both experiments, there were no systematic differences in difficulty between ToM questions and factual reasoning (i.e., noToM) questions, neither as main effects, Pilot 1: *F*(1, 27) = 1.20, *p* = .283, ${\eta}_{p}^{2}$ = .04; Pilot 2: *F*(1, 26) = 0.20, *p* = .660, ${\eta}_{p}^{2}$ = .01, nor as interactions with valence, Pilot 1: *F*(2, 54) = 1.07, *p* = .350, ${\eta}_{p}^{2}$ = .04; Pilot 2: *F*(2, 52) = 1.22, *p* = .303, ${\eta}_{p}^{2}$ = .04. However, the accuracy with which the questions were answered varied depending on the emotional valence in both experiments. Pilot 1: *F*(2, 54) = 4.49, *p* = .016, ${\eta}_{p}^{2}$ = .14; Pilot 2: *F*(2, 52) = 8.18, *p* < .001, ${\eta}_{p}^{2}$ = .24. While the questions in the neutral and negative condition (which were taken from the original EmpaToM) did not differ in their difficulty, *p*s > .129, the newly added questions were answered with a significantly higher accuracy compared to the neutral context, both in the positive condition in Pilot 1, *t*(27) = – 3.08, *p* = .015, *d* = – 0.58, and the embarrassing condition in Pilot 2, *t*(26) = – 4.19, *p* < .001, *d* = – 0.81, all other *p*s > .262, while there were no differences to the negative condition, *p*s > .264.

Beyond general analyses, we also examined the accuracy of all individual questions to identify items that were either too easy, as indicated by perfect accuracy across participants, or too difficult (or potentially misleading), as evidenced by accuracy rates falling below (or near) chance level. We identified 15 questions overall (4 too easy and 11 too difficult; see Supplement S3) and they were revised for the next phase of task evaluation.

##### Response time

When examining response times for correctly answered single-choice items, the only effect to reach significance was a main effect of valence in Pilot 1, *F*(2, 54) = 9.42, *p* < .001, η^2^ₚ = .26; all other *p*s > .270. In line with accuracy, participants responded slightly more quickly to questions following positive narrations compared to both the neutral condition, *t*(27) = 3.81, *p* = .002, *d* = 0.72, and the negative condition, *t*(27) = 2.62, *p* = .042, *d* = 0.50. In contrast, RTs did not significantly differ between the neutral and negative conditions, *t*(27) = 1.75, *p* = .273, *d* = 0.33.

**Fig. 5:**
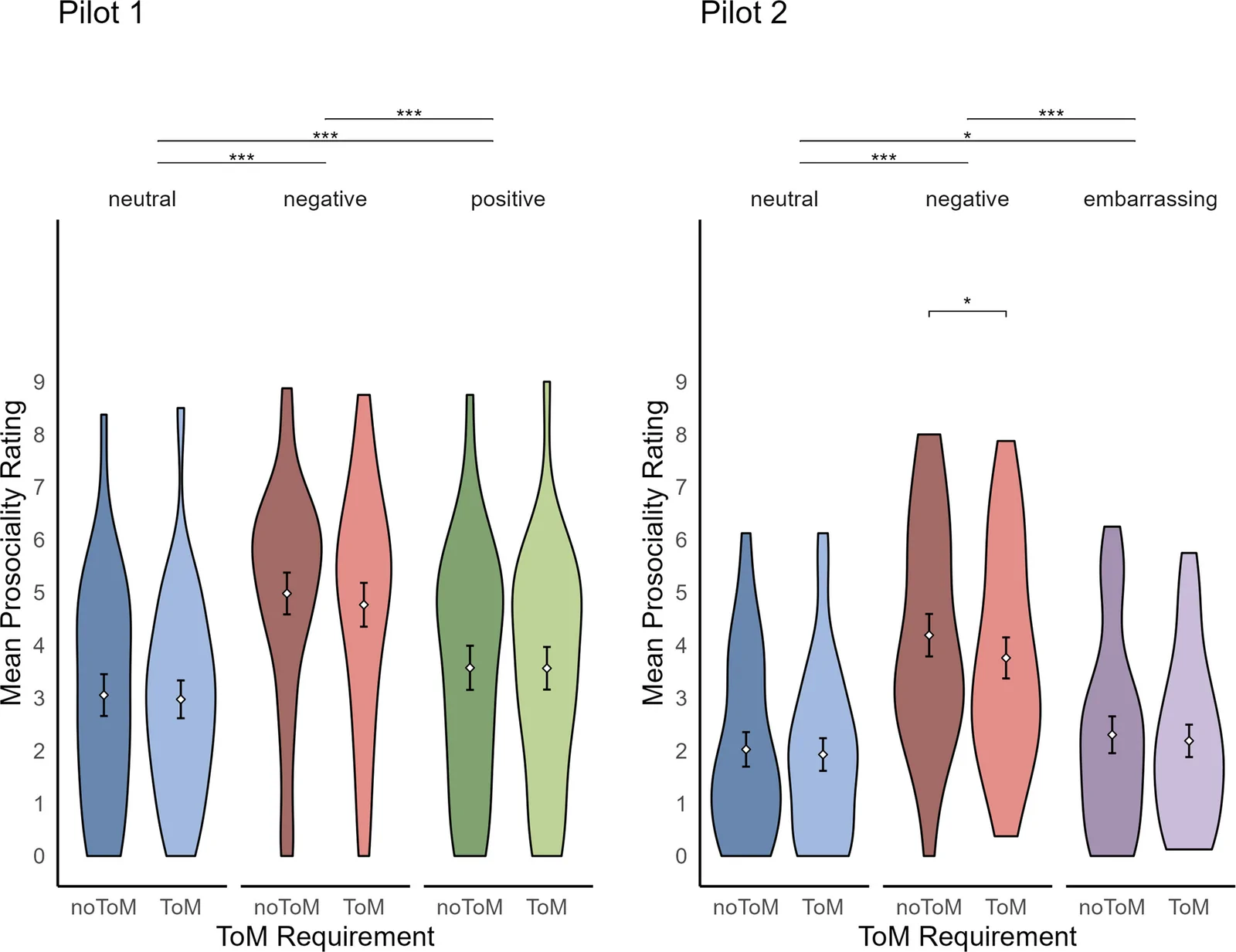
Prosociality ratings in Pilot 1 and Pilot 2. *Note.* Violin plots of mean prosociality ratings (on a scale from 0 to 9) for the single-choice questions in Pilot 1 (*left*) and Pilot 2 (*right*). *Diamonds* indicate the mean, and *error bars* represent standard errors. The *x*-axis depicts the different combinations of valence and ToM requirement. *Horizontal brackets* indicate significant differences between valence conditions (*upper brackets*) and between ToM conditions within each valence × ToM requirement combination (*lower brackets*). *p <.05, **p <.01, ***p <.001

### Prosociality rating

As expected, prosociality differed significantly between valence conditions in both Pilot 1, *F*(1.4, 38.2) = 49.42, *p* < .001, η^2^ₚ = .65, and Pilot 2, *F*(1.3, 33.3) = 68.14, *p* < .001, η^2^ₚ = .72 (see Fig. 5). Participants reported the lowest prosociality following neutral narrations and the highest prosociality following negative ones, with the newly added emotional conditions (positive and embarrassing) falling in between, all *p*s < .048. In Pilot 2, ToM requirement also had a significant effect, *F*(1, 26) = 10.26, *p* = .004, η^2^ₚ = .28, with slightly higher prosociality in the noToM condition. No other main effects or interactions reached significance in either experiment; all *ps* > .081.

## Task evaluation study

### Method

#### Sample

Participants for the task evaluation study were recruited in equal parts via the university’s recruitment platform (*n* = 38 in the final sample), the online platform Prolific (prolific.com, *n* = 38), and through private contacts (*n* = 39). They were naïve to the task (had not participated in the pilots) and received either course credit or monetary compensation for their participation. Based on a preregistered power analysis using G*Power (Faul et al., 2007), we aimed for a sample size of 62 participants per experiment, which would allow us to detect medium-sized effects. While effects of emotional valence are typically large, we also wanted sufficient power to detect relevant main effects and interactions of other factors. We initially collected data from 65 participants in Experiment 1 (positive context condition in addition to neutral and negative) and 66 participants in Experiment 2 (embarrassing context condition). We excluded four participants due to technical difficulties during the online study (e.g., videos failing to play properly). In accordance with our preregistered criteria, we also excluded six participants who reported familiarity with at least one of the narrators depicted in the videos, as well as six participants whose performance on the single-choice task was more than two standard deviations below the mean accuracy. The final sample for Experiment 1 comprised 60 participants (*M*_age_ = 31.4 years, *SD*_age_ = 10.7; 60% female, 2% diverse), and the final sample for Experiment 2 included 55 participants (*M*_age_ = 29.3 years, *SD*_age_ = 11.1; 60% female).

#### Study material and procedure

As in the pilot study, we used sets of eight narrators, each contributing eight videos. We retained six of the sets from the pilot and re-recorded the remaining two (‘Klara’ and ‘Tobias’), as they had either failed to elicit the expected emotional responses or were perceived as inauthentic based on participant feedback. In addition, we revised the response options for 15 comprehension questions that had been identified as unsuitable, either because they were too easy, overly difficult, or potentially misleading. We again ensured that the presented options did not systematically differ between conditions in terms of key linguistic features (word count, use of the subjunctive mood, passive constructions). Other than these adjustments, the study procedures were kept consistent with the pilot phase (see Fig. 1).

#### Design and analysis

As in the pilot study, we conducted 3 × 2 repeated-measures ANOVAs for each experiment with the within-subject factors valence (neutral, negative, positive/embarrassing [Exp. 1/Exp. 2]) and ToM requirement (noToM, ToM). In cases where the assumption of sphericity was violated, we applied Greenhouse-Geisser corrections. Post-hoc comparisons were performed using Bonferroni-corrected *t* tests. We analyzed the following dependent variables: (i) affect ratings, with empathic responding operationalized as the difference between neutral and emotional narrations; (ii) accuracy on the comprehension questions; (iii) response times for correctly answered questions; (iv) prosociality, measured by the willingness to invest hypothetical resources to benefit the narrator; and (v) self-reported embarrassment (assessed in Experiment 2 only).

In addition to the ANOVAs, we explored interindividual associations between our key dependent variables. First, to probe the relation between empathic responding, ToM performance, and prosociality (see Kanske et al., 2016; Lehmann et al., 2022), we computed participant-level means for the negative empathic responding (operationalized as the affect rating difference between negative and neutral trials), response accuracy, and prosociality. Using these aggregate scores, we examined whether we could replicate prior results, namely that negative empathic responding is independent of ToM accuracy but related to greater prosocial behavior.

To investigate the relation between empathic responding in the novel emotional contexts to negative empathy, to ToM performance, and to prosociality, we additionally calculated a positive empathy score (affect rating difference between positive and neutral trials) in Experiment 1 and a measure of empathic embarrassment (embarrassment difference between embarrassing and neutral trials) in Experiment 2. We then assessed whether individual differences in negative empathic responding were related to positive empathy (in Experiment 1) and empathic embarrassment (in Experiment 2). Furthermore, we probed the relation between positive/embarrassment empathy with ToM performance and prosocial behavior.

### Results

#### Affect rating

**Fig. 6:**
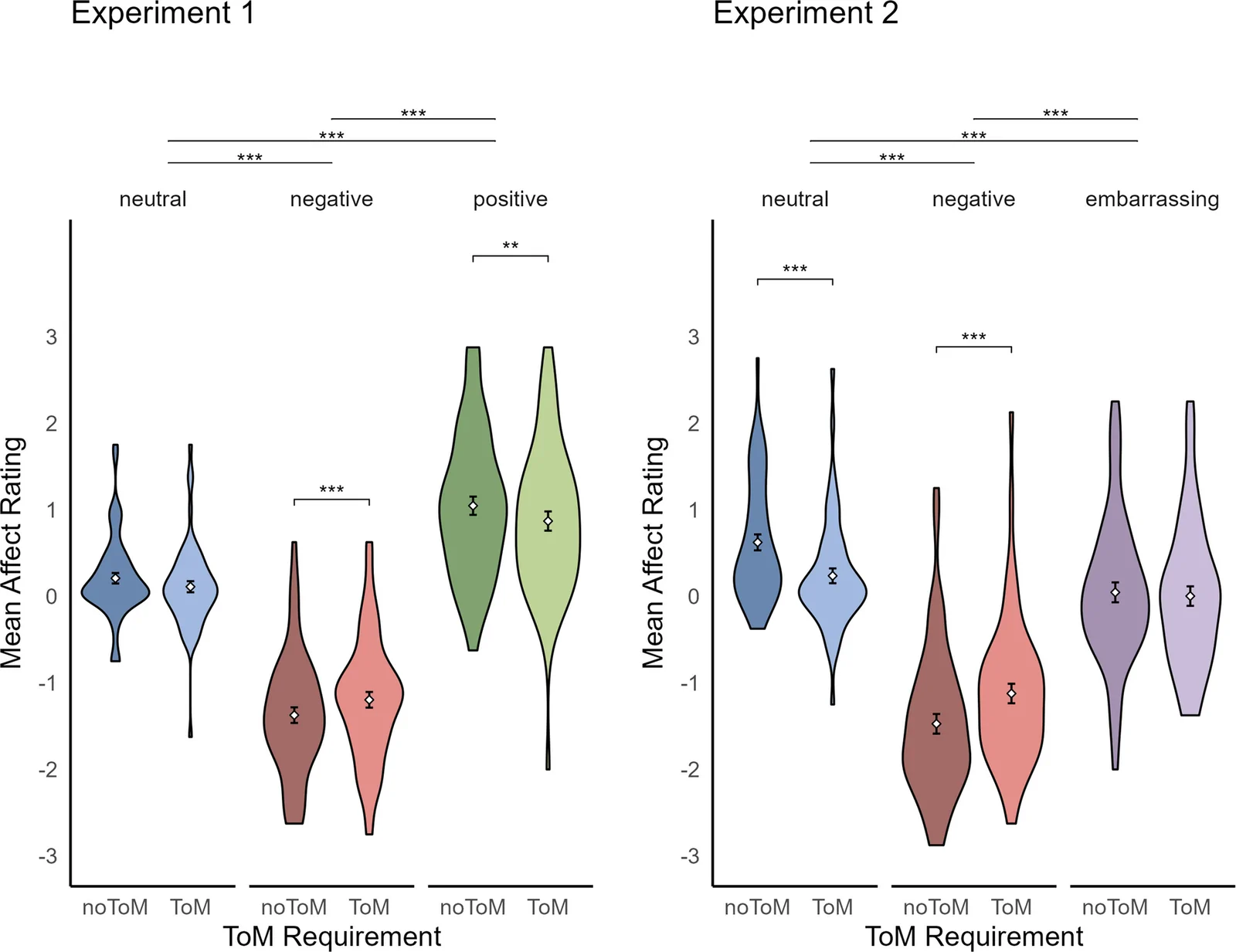
Affect ratings in Experiment 1 and Experiment 2. *Note.* Violin plots of mean affect ratings (on a scale from – 3 to + 3) in Experiment 1 (*left*) and Experiment 2 (*right*). *Diamonds* indicate the mean, and *error bars* represent standard errors. The *x*-axis depicts the different combinations of valence and ToM requirement. *Horizontal brackets* indicate significant differences between valence conditions (*upper brackets*) and between ToM conditions within each valence × ToM requirement combination (*lower brackets*). *p <.05, **p <.01, ***p <.001

Figure 6 displays affect ratings for both experiments in the evaluation study. Participants’ affect varied significantly with the emotional valence of the narration in both experiments, Exp. 1: *F*(1.1, 64.3) = 174.68, *p* < .001, ${\eta}_{p}^{2}$ = .75; Exp. 2: *F*(2, 108) = 192.06, *p* <.001, ${\upeta}_{\mathrm{p}}^{2}$ = .78. Consistent with predictions, participants reported lower affect following negative narrations compared to neutral narrations, Exp. 1: *t*(59) = 13.87, *p* < .001, *d* = 1.79; Exp. 2: *t*(54) = 16.35, *p* < .001, *d* = 2.20. In Experiment 1, the positive condition yielded significantly higher affect than both the negative condition, *t*(59) = – 13.41, *p* < .001, *d* = – 1.73, and the neutral condition, *t*(59) = – 10.70, *p* < .001, *d* = – 1.38. In Experiment 2, the embarrassing condition produced lower affect than the neutral condition, *t*(54) = 4.94, *p* < .001, *d* = 0.67, though affect remained higher than in the negative condition, *t*(54) = – 15.17, *p* < .001, *d* = – 2.05.

While ToM requirement did not have a main effect on participants’ affect, Exp. 1: *F*(1, 59) = 1.34, *p* = .252, ${\eta}_{p}^{2}$ = .02; Exp. 2: *F*(1, 54) = 0.78, *p* = .381, ${\eta}_{p}^{2}$ = .01, significant interactions between valence and ToM requirement emerged in both experiments, Exp. 1: *F*(2, 118) = 14.61, *p* < .001, ${\eta}_{p}^{2}$ = .20; Exp. 2: *F*(2, 108) = 38.45, *p* < .001, ${\eta}_{p}^{2}$ = .42. In the negative condition, participants reported lower affect following noToM narrations compared to ToM narrations, Exp. 1: *t*(59) = – 4.38, *p* < .001, *d* = – 0.57; Exp. 2: *t*(54) = – 7.58, *p* <.001, *d* = – 1.02. The neutral condition showed the opposite (descriptive) pattern, with higher affect following noToM than ToM narrations. This difference was significant in Experiment 2, *t*(54) = 5.54, *p* <.001, *d* = 0.75, but not in Experiment 1, *t*(59) = 1.95, *p* = .168, *d* = 0.25. In the newly added positive condition, a similar pattern was observed, with noToM narrations eliciting slightly higher affect than ToM narrations, *t*(59) = 3.25, *p* = .006, *d* = 0.42. In the embarrassing condition, no difference in affect emerged between ToM and noToM narrations, *t*(54) < 1.

#### Embarrassment rating

**Fig. 7:**
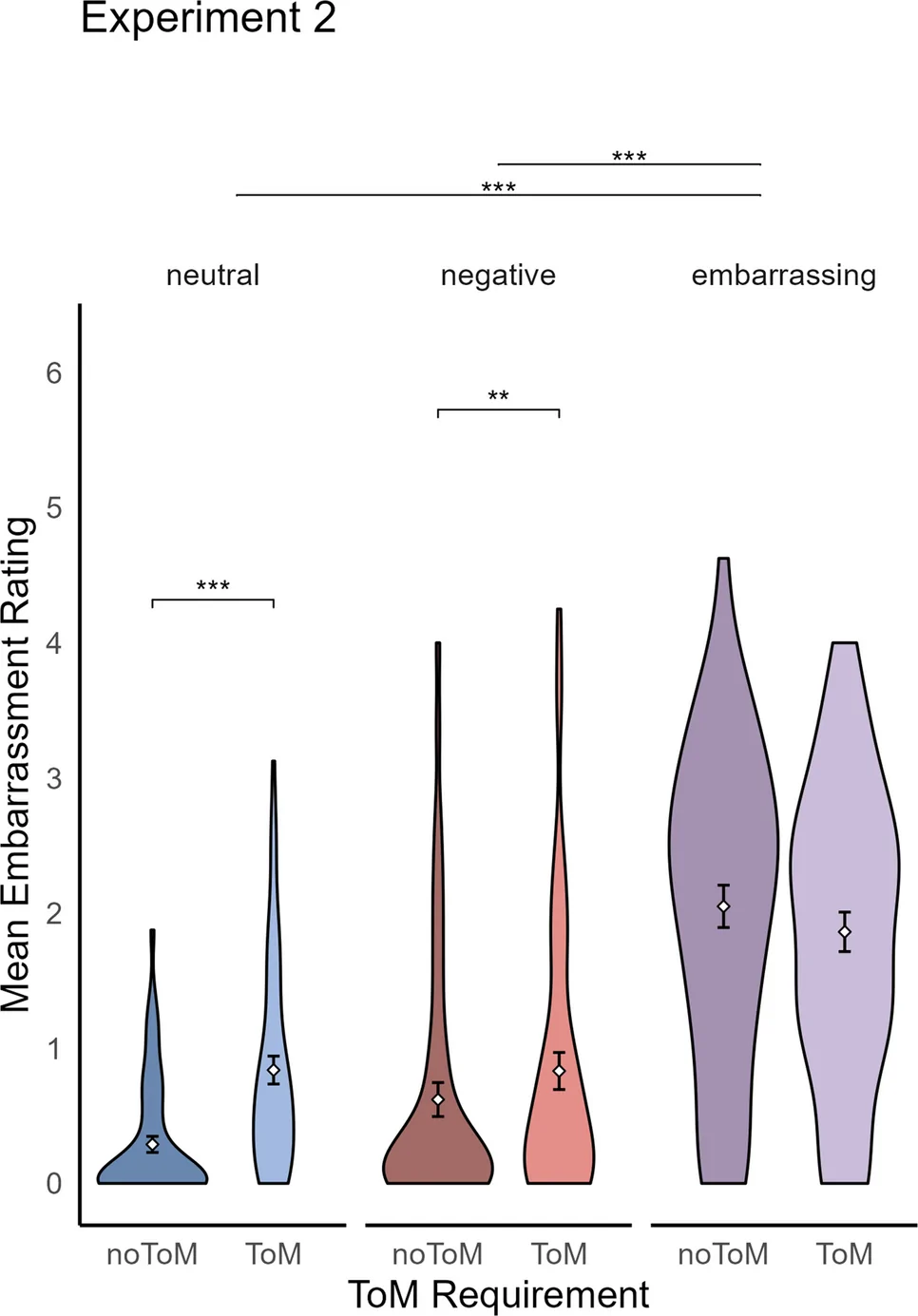
Embarrassment ratings in Experiment 2. *Note.* Violin plot of mean embarrassment ratings (on a scale from 0 to 6) in Experiment 2. *Diamonds* indicate the mean, and *error bars* represent standard errors. The *x*-axis depicts the different combinations of valence and ToM requirement. *Horizontal brackets* indicate significant differences between valence conditions (*upper brackets*) and between ToM conditions within each valence × ToM requirement combination (*lower brackets*). *p <.05, **p <.01, ***p <.001

In Experiment 2, participants were additionally asked to rate how embarrassed they felt after listening to each narration (see Fig. 7). Embarrassment ratings varied significantly depending on the valence of the narration, *F*(1.7, 93.1) = 58.25, *p* < .001, η^2^ₚ = .52. As expected, participants reported greater embarrassment following embarrassing narrations compared to both the neutral condition, *t*(54) = − 10.40, *p* < .001, *d* = − 1.40, and the negative condition, *t*(54) = − 7.41, *p* < .001, *d* = − 1.00. There was no significant difference in embarrassment ratings between the neutral and negative narrations, *t*(54) = − 1.36, *p* = .534, *d* = − 0.18.

Beyond valence, ToM requirement also influenced embarrassment ratings, as indicated by a significant main effect, *F*(1, 54) = 20.87, *p* < .001, η^2^ₚ = .28, and a significant interaction with valence, *F*(1.7, 92.9) = 29.28, *p* < .001, η^2^ₚ = .35. Specifically, participants reported feeling somewhat more embarrassed after ToM than noToM narrations in both the neutral condition, *t*(54) = – 8.92, *p* < .001, *d* = – 1.20, and the negative condition, *t*(54) = – 3.22, *p* = .006, *d* = – 0.43, while ToM requirement had no significant effect in the embarrassing condition, *t*(54) = 2.35, *p* = .069, *d* = 0.32.

#### Performance in single-choice task

As in the pilot experiments, we examined performance on the single-choice questions to assess question difficulty and potential systematic differences between conditions.

##### Accuracy

**Fig. 8:**
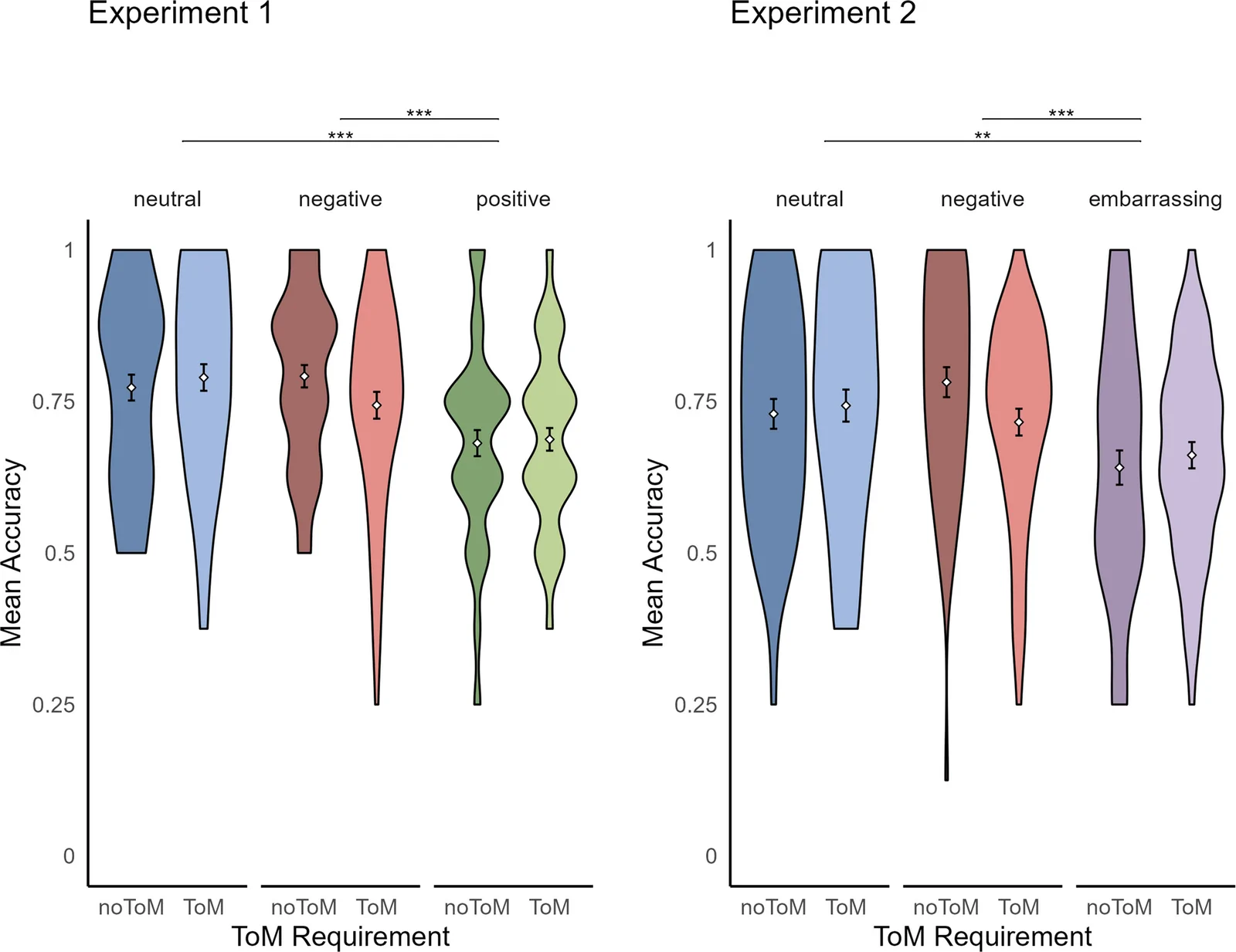
Accuracy in Experiment 1 and Experiment 2. *Note.* Violin plots of mean accuracy (from 0 to 1) in the single-choice questions in Experiment 1 (*left*) and Experiment 2 (*right*). *Diamonds* indicate the mean, and *error bars* represent standard errors. The *x*-axis depicts the different combinations of valence and ToM requirement. *Horizontal brackets* indicate significant differences between valence conditions (*upper brackets*) and between ToM conditions within each valence × ToM requirement combination (lower brackets). *p <.05, **p <.01, ***p <.001

Accuracy results of both experiments in the task evaluation study are displayed in Fig. 8. Participants’ accuracy on the single-choice questions did not differ based on whether the question required ToM or factual reasoning, as indicated by a non-significant main effect of ToM requirement, *F*s < 1, and non-significant interactions with valence, Exp. 1: *F*(2, 118) = 1.67, *p* = .193, η^2^ₚ = .03, Exp. 2: *F*(2, 108) = 2.56, *p* = .082, η^2^ₚ = .04. However, accuracy differed significantly across valence conditions: Exp. 1, *F*(2, 118) = 17.39, *p* < .001, η^2^ₚ = .23; Exp. 2, *F*(2, 108) = 10.78, *p* < .001, η^2^ₚ = .17. While, similar to the pilot experiments, there were no significant differences between neutral and negative trials in either experiment, *ts* < 1, accuracy was slightly lower in the newly added valence conditions, contrary to the findings of the pilot study. In Experiment 1, questions in the positive condition were answered with reduced accuracy compared to both the neutral, *t*(59) = 5.48, *p* < .001, *d* = 0.71, and negative conditions, *t*(59) = 4.54, *p* < .001, *d* = 0.59. Similarly, in Experiment 2, accuracy in the embarrassing condition was slightly lower than in the neutral, *t*(54) = 3.64, *p* = .002, *d* = 0.49, and negative conditions, *t*(54) = 4.15, *p* < .001, *d* = 0.56.

##### Response time

RTs for correctly answered questions varied across valence conditions in both experiments, Exp. 1: *F*(2, 118) = 5.07, *p* = .008, η^2^ₚ = .08; Exp. 2: *F*(1.8, 96.2) = 7.93, *p* = .001, η^2^ₚ = .13. In Experiment 1, RTs were notably longer for neutral trials than for positive, *t*(59) = 3.14, *p* =.009, *d* = 0.41, or negative trials, *t*(59) = 2.55, *p* = .039, *d* = 0.33. A similar effect was observed in Experiment 2, where neutral trials produced slower responses than embarrassing trials, *t*(54) = 4.69, *p* < .001, *d* = 0.63, all other *p*s > .159. Additionally, there was a significant main effect of ToM requirement in Experiment 1, *F*(1, 59) = 5.20, *p* = .026, η^2^ₚ = .08, indicating that participants took slightly longer to respond correctly to ToM questions compared to noToM questions. No other main effects or interactions reached significance; all *p*s >.280. To assess whether extreme values influenced these results, we applied a stricter exclusion criterion, removing RTs exceeding 60 s. Reanalyzing the data under this criterion did not alter the pattern of results.

##### Prosociality rating

**Fig. 9:**
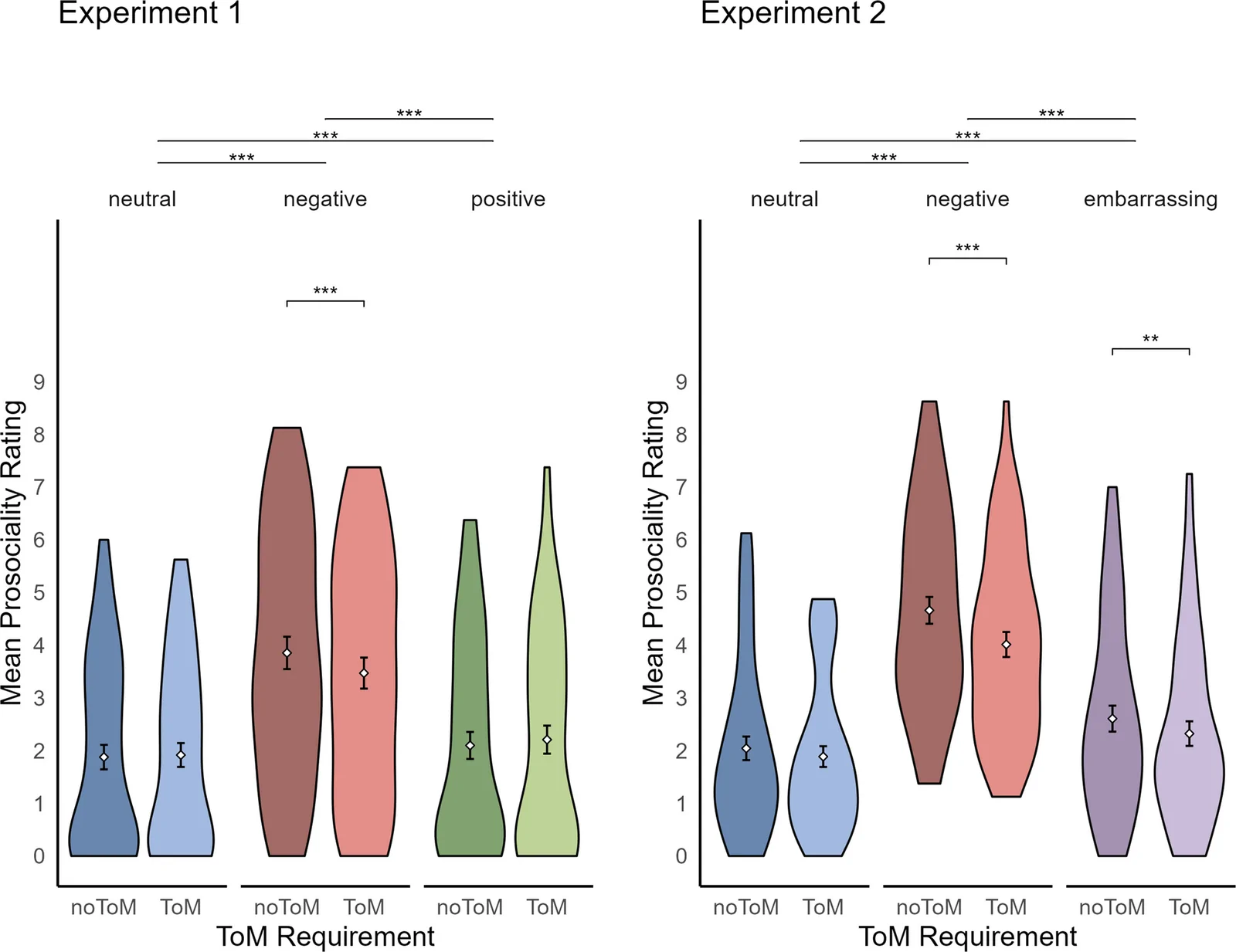
Prosociality ratings in Experiment 1 and Experiment 2. *Note.* Violin plots of mean prosociality ratings (on a scale from 0 to 9) for the single-choice questions in Experiment 1 (*left*) and Experiment 2 (*right*). *Diamonds* indicate the mean, and *error bars* represent standard errors. The *x*-axis depicts the different combinations of valence and ToM requirement. *Horizontal brackets* indicate significant differences between valence conditions (*upper brackets*) and between ToM conditions within each valence × ToM requirement combination (*lower brackets*). *p <.05, **p <.01, ***p <.001

Participants’ willingness to invest resources on behalf of the narrator varied significantly depending on the emotional valence of the narration, Exp. 1: *F*(1.2, 70.4) = 85.89, *p* < .001, η^2^ₚ = .59; Exp. 2: *F*(1.7, 91.1) = 138.39, *p* < .001, η^2^ₚ = .72 (see Fig. 9). Consistent with previous research, prosociality was substantially higher in the negative condition compared to the neutral condition, Exp. 1: *t*(59) = – 10.67, *p* < .001, *d* = – 1.38; Exp. 2: *t*(54) = – 13.96, *p* < .001, *d* = – 1.88. As in pilot experiments, the newly added valence conditions (positive and embarrassing) fell between the neutral and negative conditions in terms of the prosocial responses they elicited. Specifically, participants reported greater willingness to help following positive and embarrassing narrations compared to neutral ones, Exp. 1: *t*(59) = – 4.05, *p* < .001, *d* = – 0.52; Exp. 2: *t*(54) = – 4.37, *p* < .001, *d* = – 0.59, but still lower willingness to help compared to the negative condition, Exp. 1: *t*(59) = 8.41, *p* < .001, *d* = 1.09; Exp. 2: *t*(54) = 11.64, *p* < .001, *d* = 1.57.

As indicated by significant valence × ToM requirement interactions, Exp. 1: *F*(2, 118) = 13.94, *p* < .001, η^2^ₚ = .19; Exp. 2: *F*(2, 108) = 9.67, *p* < .001, η^2^ₚ = .15, as well as significant main effects of ToM requirement, Exp. 1: *F*(1, 59) = 5.06, *p* = .028, η^2^ₚ = .08; Exp. 2: *F*(1, 54) = 37.12, *p* < .001, η^2^ₚ = .41, ToM requirement modulated the effect of valence on prosociality. Specifically, noToM narrations elicited higher prosociality ratings than ToM narrations in the negative condition of both experiments, Exp. 1: *t*(59) = 4.94, *p* < .001, *d* = 0.64; Exp. 2: *t*(54) = 6.24, *p* < .001, *d* = 0.84, as well as in the embarrassing condition of Experiment 2, *t*(54) = 3.41, *p* = .003, *d* = 0.46. By contrast, ToM requirement had no significant effect on prosociality in the neutral condition, Exp. 1: *t*(59) < 1; Exp. 2: *t*(54) = 1.97, *p* = .162, *d* = 0.27, or in the positive condition of Experiment 1, *t*(59) = – 1.80, *p* = .234, *d* = – 0.23.

#### Interindividual correlations

In addition to our main analyses, we examined correlations between participants’ mean scores for negative empathic responding (affect difference between negative and neutral trials), positive empathic responding (affect difference between positive and neutral trials; Experiment 1 only), empathic embarrassment (embarrassment difference between embarrassing and neutral trials; Experiment 2 only), ToM performance (response accuracy), and prosociality. A complete overview of the correlations from both the pilot studies and task evaluation experiments is provided in Supplement S4.

We first examined associations between empathic resonance and both ToM performance and prosociality within each emotional context, as previous research using the EmpaToM revealed independence of empathy and ToM tendencies on an inter-individual level (see Kanske et al., 2015, 2016), but pointed towards a positive contribution of empathy (and slightly also ToM) to prosociality (Lehmann et al., 2022).

Across experiments, negative empathic responding was not significantly related to overall ToM accuracy (Exp. 1: *r*(58) = .04, *p* = .737; Exp. 2: *r*(53) = .23, *p* = .095). The link between negative empathy and prosociality differed across experiments: In Experiment 1, stronger negative empathy was associated with higher prosociality overall (*r*(58) = .36, *p* = .005) and specifically for negative narrations (*r*(58) = .54, *p* < .001), whereas in Experiment 2 these correlations were numerically present, but nonsignificant (prosociality overall: *r*(53) = .22, *p* = .111; prosociality in negative trials: *r*(53) = .21, *p* = .119). Positive empathic responding (Experiment 1) showed a similar pattern to negative empathy, correlating with greater prosociality (both overall: *r*(58) = .47, *p* < .001, and for positive trials: *r*(58) = .40, *p* = .002), but not with ToM accuracy (*r*(58) = –.05, *p* = .731). In contrast, in Experiment 2, empathic embarrassment was unrelated to prosociality (prosociality overall: *r*(53) = .01, *p* = .933; prosociality in embarrassing trials: *r*(53) = .01, *p* = .921) but slightly positively associated with ToM accuracy (*r*(53) = .27, *p* = .048). No significant correlations between ToM accuracy and prosociality were observed in either experiment (Exp. 1: *r*(58) = .15, *p* = .250; Exp. 2: *r*(53) = -.06, *p* = .652).

Finally, we examined whether different empathy types were interrelated. In Experiment 1, negative and positive empathy were strongly correlated (*r*(58) = .74, *p* < .001). In Experiment 2, negative empathy was also positively correlated with empathic embarrassment (*r*(53) = .56, *p* < .001).

## General discussion

To understand how people resonate with and mentalize about others, researchers need to investigate the mechanisms and effects of empathy and theory of mind (ToM) in different experimental setups and varying contexts. The current studies focus on *emotional* context, establishing and testing two extended versions of the EmpaToM task – a video-based measure of social affect, cognition and behavior (Kanske et al., 2015; Lehmann et al., 2022) – by adding positive and embarrassing video stimuli to the existing neutral and negative videos.

Specifically, we chose eight sets from the original EmpaToM, each of which constituted four videotaped narrations of a fictitious character according to the factors emotional valence of their narration (so far: neutral vs. negative) and ToM requirement of their narration and subsequent question (noToM vs. ToM). Building on these sets, two positive (EmpaToM-pos) and two embarrassing narrations (EmpaToM-embarrass) were developed for each set (one for each ToM condition; including the respective questions and response options), resulting in three levels of emotional valence in each task. We created new videos for all conditions and tested the new positive and embarrassing videos with neutral and negative videos in two respective pilot experiments (Pilot 1: negative, neutral, positive; Pilot 2: negative, neutral, embarrassing). Finally, Experiments 1 and 2 (Experiment 1: neutral, negative, positive; Experiment 2: neutral, negative, embarrassing) probed the improved sets that were adjusted based on insights from the pilots (e.g., to enhance video quality and alter question difficulty).

In short, both expanded EmpaToM tasks successfully induced empathy according to the novel valence conditions, allowed us to measure ToM performance at typical levels and without indications of a ceiling effect, and provided insights into the relation between empathy, ToM performance, and prosociality within and across neutral, negative, positive, and embarrassing emotional contexts. We briefly summarize and discuss each of these findings in the subsequent sections before deriving more general suggestions and conclusions.

### Social understanding and behavior in positive and embarrassing emotional contexts

Results of all experiments demonstrate that the novel videos effectively induced empathy in participants, according to the intended emotion: Participants reported substantially higher positive affect after positive narrations compared to neutral and negative narrations (Pilot 1 and Experiment 1) and rated their embarrassment level higher after watching embarrassing than after watching neutral and negative videos, indicating empathic embarrassment (Pilot 2 and Experiment 2). These patterns were revealed in summary statistics as well as within all final individual sets (see Supplement S2). Also, participants, after watching embarrassing videos, felt slightly worse than after neutral videos, but still better than after negative videos (Experiment 2). This finding suggests that the EmpaToM setup did not elicit schadenfreude in participants watching embarrassing videos presumably because the situation lacked factors that typically favor schadenfreude, such as competitiveness (e.g., Steinbeis & Singer, 2013) or serious wrongdoing by the narrator (e.g., Smith et al., 2009). Prior research suggests that whether or not embarrassment is explicitly rated influences the relative elicitation of schadenfreude versus empathic embarrassment (Paulus et al., 2018). Retaining both ratings in the EmpaToM-embarrass therefore seems advisable, as their combination may help clarify the distinct processes and influencing factors underlying each emotion. In both the EmpaToM-pos and EmpaToM-embarrass, original findings on empathy for negative narrations were replicated (Kanske et al., 2015, 2016): Participants reported more negative affect after negative compared to neutral narrations, an effect that was somewhat stronger in noToM than ToM videos (Experiments 1 and 2). Similarly, affect ratings for positive videos were slightly higher in the noToM compared to the ToM condition. As with negative valence, the noToM narrations may convey and elicit positive affect more purely and directly, while mentalizing requirements could dampen emotion processing and empathic resonance, in line with studies reporting a down-regulation of (both negative and positive) emotional responses under high cognitive load (e.g., Barley et al., 2021; Erk et al., 2007). Ratings of the participants’ embarrassment, by contrast, yielded somewhat higher values for ToM narrations. This pattern is likely due to the nature of the stories, as ToM requirements are often implemented by narrators using ironic remarks, vague hints, or insinuations, which may induce minor discomfort or awkwardness given these kinds of utterances can deviate somewhat from social norms (Keltner, 1996; Tangney et al., 1996; Weisfeld & Weisfeld, 2014).

Performance on the questions requiring ToM and factual reasoning replicated previous findings (Breil et al., 2021; Kanske et al., 2015), suggesting that the task, while being feasible overall, is sufficiently challenging to induce inter-individual variance in ToM performance in healthy adult samples, which is not the case in many standard ToM measures (Brunet et al., 2000; Happé, 1994; Wimmer & Perner, 1983). In addition, ToM and factual reasoning questions were of similar difficulty in both experiments and for both new emotion conditions, which is critical for fMRI studies that compare activation patterns during mentalizing with an optimally matched control condition. However, after adjustments based on pilot results, performance in the new emotion conditions was worse overall compared to the neutral and negative conditions (average accuracy of 68%/65% for positive and embarrassing stimuli, respectively, compared to an average accuracy of around 75% for neutral and negative videos). Analyzing all sets individually clarified which items could be adjusted or selected in studies that require similar difficulty levels for all emotional valence conditions (see Supplement S3).

As in previous studies, participants reported higher willingness to act prosocially towards the person in the video after hearing them recount negative compared to neutral narrations (Lehmann et al., 2022). Both joyful and embarrassing narrations were accompanied by lower prosociality towards the narrator compared to negative narrations but led to higher prosociality compared to neutral narrations. Joyful narrations may have increased prosociality (compared to the neutral condition) by inducing positive affect sharing, an interpretation that is supported by their correlation (see below; Andreychik & Migliaccio, 2015; Depow et al., 2021). Specifically, some researchers have argued that the positive emotional state that is elicited in the observer when witnessing someone else being happy may, in turn, motivate the observer to engage in prosocial behavior to prolong and maintain their own current positive affect (mood maintenance hypothesis; e.g., Telle & Pfister, 2012, 2016). For embarrassment, enhanced generosity (compared to the neutral condition) is in line with the idea that the expression of feeling embarrassed can signal an individual’s care to comply with social norms and thereby foster social approach towards them (Keltner, 1996; Tangney et al., 1996). Similar to empathic resonance, and likely due to comparable reasons, generosity was slightly higher after videos that did not involve mentalizing in the negative and embarrassing context.

When examining the relationships between our variables across individuals, we found that empathic resonance for negative narrations was strongly and positively correlated with empathic resonance for positive narrations (positive empathy) and with empathic resonance for embarrassing narrations (empathic embarrassment). This pattern suggests that individuals’ tendency to empathize generalizes across different emotional contexts. By contrast, and in line with the literature, the tendency to empathize with the person in the video (affect rating difference between emotional and neutral videos) was not related to mentalizing performance for either negative or positive emotions (Kanske et al., 2016; Weisz & Cikara, 2021). In contrast, stronger empathic responses to embarrassing narrations (Experiment 2) were linked to slightly higher theory of mind performance. This effect is small and may well be a random finding. However, given embarrassment is a more complex and especially social emotion, empathic resonance and mentalizing may benefit each other in this context.

With regard to prosocial behavior, we found no evidence that mentalizing ability increased participants’ willingness to help (Lehmann et al., 2022). Higher empathic resonance, however, was associated with higher prosociality in the negative emotional context and also in the newly introduced positive context. Notably, we did not observe a similar effect for empathic embarrassment, which may suggest that this emotional state elicits different behavioral motivations. While some researchers argue that signaling embarrassment can enhance the chance for social approach (e.g., Feinberg et al., 2012), it clearly creates a more ambivalent context in which avoidance and approach motives may be in conflict. Thus, despite substantial similarities in social understanding across emotional contexts, there appear to be specific differences (particularly in empathy) that warrant further investigation.

### Applications, limitations, and conclusions

We think our novel tasks are suitable to investigate empathy, ToM, and prosociality in realistically dynamic, yet experimentally controlled settings involving different and relevant emotional contexts. Specifically, the EmpaToM-pos provides a tool to address the processing and response to observing joy in others, complementing the hitherto strong focus on negative valence (Depow et al., 2021; Singer & Lamm, 2009) and allowing direct comparisons of positive with negative and neutral settings by employing a variety of joy-related narrations (e.g., happiness about a new relationship, looking forward to a vacation, enjoyment of a hobby, or professional success). The EmpaToM-embarrass targets the understanding and responding to an ambivalent, complex, and distinctly social emotion (Edelmann, 2012; Keltner, 1996; Tangney et al., 1996) across a variety of incidents (e.g., narrations about throwing up during a date, spilling coffee in a nice café, or having forgotten your wallet while standing at the checkout) and provides, to our knowledge, the first tool to directly compare empathic embarrassment to empathic resonance in negative and neutral contexts. As the EmpaToM provides valid measures of empathy, ToM, and prosocial behavior as well as their neural underpinnings in adult populations (Kanske et al., 2015; Lehmann et al., 2022; Tholen et al., 2020), the expanded versions can foster the understanding of similarities, relations, and differences between these processes in distinct emotional setups (Andreychik & Migliaccio, 2015; Morelli et al., 2014).

Beyond generalizing – or distinguishing – empathy and mentalizing across valences, a critical question in social cognition research pertains to the interrelation of empathy and ToM (Weisz & Cikara, 2021). Studies reported the absence of a relation between empathy and ToM on an inter-individual level (empathizers are not necessarily good or bad mentalizers), but dynamic causal modeling suggested an intricate relationship in that acute empathizing during negative narrations can hamper successful mentalizing (Kanske et al., 2016). Employing the extended EmpaToM tasks in fMRI setups would allow us to probe the nature of this relation in positive and embarrassing settings. Furthermore, while our everyday lives are filled with opportunities for social affect, cognition, and behavior (Depow et al., 2021; Ho et al., 2022), people seem to differ in the extent to which they engage in these (mental) activities (e.g., Stietz et al., 2019; Thielmann et al., 2020). Measuring empathy, ToM, and generosity across emotional contexts may provide a more valid account of individual tendencies and more reliably identify prerequisites, related constructs, and consequences of inter-individual differences.

The EmpaToM-pos and EmpaToM-embarrass paradigms offer considerable flexibility and can be adapted to suit different purposes. Researchers using the tasks are free to omit the prosociality rating if it is of no interest to them or to include additional measures such as the established compassion ratings (“How much compassion do you feel for the person in the video?”) and metacognition estimates (“How confident are you about your answer?”, rated after the factual reasoning/ToM questions; Kanske et al., 2015, 2016; Molenberghs et al., 2016). Although the new EmpaToM versions include fewer trials per condition than the original paradigm, we were able to reproduce well-established effects, consistent with prior evidence that reduced trial numbers can still yield stable behavioral and fMRI results (e.g., Tholen et al., 2020). Further shortening may be desirable depending on the research context, particularly in studies with lengthy procedures or extensive test batteries. In non-neuroimaging settings, additional simplifications, such as reducing the number of conditions, may be feasible: For instance, the noToM condition could be omitted if the study focuses exclusively on moderators of mentalizing or interindividual differences and does not require factual reasoning as a comparison condition. Similarly, a neutral baseline condition may not always be necessary (given appropriate counterbalancing across participants) if the research question concerns relative differences between experimental manipulations (e.g., whether participants are more emotionally affected in one condition compared to another). Such adaptations would substantially reduce the number of trials required to assess empathy and ToM. This may also make it feasible to include both positive and embarrassing conditions within a single study, which were examined separately in the present work to keep the overall trial number manageable for participants.

The present experiments and tasks are not without limitations. For once, the overall difference in accuracy rates between the established neutral/negative and the novel valence conditions will require researchers who need comparable difficulties in all conditions to either select individual sets according to their demands or adjust some of the sets to alter item difficulty. While this overall difference does not matter to the exemplary research questions outlined above, the EmpaToM may not be suitable to study whether distinct emotions have differential effects on mentalizing success per se. In the Supplements, we provide the difficulty level for each single-choice question (S3), along with the full text of all narrations and questions to facilitate adaptation. We also include suggested selections of speaker sets (reduced from eight to six narrators) for both the EmpaToM-pos and EmpaToM-embarrass, that are balanced in difficulty across valence conditions (S5). In addition, it should be noted that the present experiments demonstrate the successful implementation of positive and embarrassing contexts but were not designed to provide normative data on empathy tendency, mentalizing performance, or generosity. They also do not include direct tests of convergent validity through comparisons with external measures, although the findings offer initial support for construct validity, as reflected in successful empathy induction, and for nomological validity, indicated by the expected association between empathy and prosocial behavior. In addition, the reported paradigm does not allow for inferences about the specific stimulus features driving empathic responding, as the videos are multimodal and include both verbal and non-verbal cues. For the original EmpaToM, prior studies from our lab have shown that comparable levels of empathy and mentalizing can be elicited when only the audio track is presented, suggesting that empathic responses are not solely or mainly driven by the narrator’s emotional facial expressions (Landmann et al., 2024, 2025). However, for the adapted versions introduced here, systematic investigations would be required to make a similar claim, as it is conceivable that emotional facial expressions play a more central role in positive or embarrassing contexts than in negative ones. Finally, and like the original, the novel EmpaToMs are heavily language-based and therefore not suitable for children.

To broaden the applicability of the task and deepen our understanding of social affect and cognition, future endeavors could involve the creation of parallel sets and English versions for the extended paradigm, allowing the investigation of intervention effects and retest stability in more representative samples. Additionally, a range of emotional valences should be incorporated or differentiated further: For instance, when implementing negative valence, the EmpaToM and comparable tasks often employ a wide and intermixed range of emotional timbres, amongst others, sadness, despair, anxiety, distress, or even anger. Addressing similarities and differences in empathy, mentalizing, and prosocial tendencies across and between these negatively valenced states would require including the respective emotional contexts in well-construed distinctive stimuli. The same applies to positive emotions, where distinct states like joy and pride may elicit different observer responses. Likewise, many studies emphasize the difference between the social emotions embarrassment, guilt, and shame (e.g., Edelmann, 2012; Keltner, 1996; Miller, 1995; Tangney et al., 1996), which could be further examined concerning their (similar or differential) effects on social understanding and behavior.

To summarize, we offer German versions of two extended EmpaToM tasks that include positive and embarrassing in addition to neutral and negative narrations. Two pilots (*N*s = 28/27) and two stronger-powered experiments (*N*s = 60/55) demonstrate their efficiency in inducing empathy and prosociality and in measuring ToM without a ceiling effect. Initial findings point towards similarities as well as differences in empathy, ToM, and their contribution to interpersonal decision making in distinct emotional contexts.

## Supplementary Information

Below is the link to the electronic supplementary material.

Supplementary file1 (PDF 367 KB)

## Data Availability

Data are available on PsychArchives (pilot: https://doi.org/10.23668/psycharchives.22644; task validation: https://doi.org/10.23668/psycharchives.22645).

## Footnotes

1 Note that the EmpaToM also allows the assessment of compassion as the caring, warm feeling *for* another (Kanske et al., 2015).

## Code availability

Code is available on PsychArchives (https://doi.org/10.23668/psycharchives.22646).
