# Supplementary material for: The good, the bad, and the cringy: Introducing the EmpaToM-pos and EmpaToM-embarrass as tools for assessing empathy and theory of mind in diverse emotional contexts
Source: Behav Res Methods. 2026 Nov 2;58(12):339. doi: 10.3758/s13428-026-03202-x (PMC13727813; doi:10.3758/s13428-026-03202-x)
Supplement: Supplementary file 1 [file 13428_2026_3202_MOESM1_ESM.pdf]

Supplementary Materials for

The good, the bad, and the cringy: Introducing the EmpaToM-pos and EmpaToM-embarrass as tools to assess empathy and Theory of Mind in diverse emotional contexts

Eva Landmann[1*] and **Anne Böckler**[1]

[1]Julius-Maximilians-Universität Würzburg, Department of Psychology, Würzburg, 97070, Germany

*eva.landmann@uni-wuerzburg.de

Content

S1 Example Sets of Narrations

S2 Empathy Induction in Individual Sets

S3 Question Accuracy in Single-Choice Questions Per Narration

S4 Inter-Individual Correlations

S5 Suggested Set Selection for Balanced Difficulty

S1 Example Narrations

For this study, we expanded existing stimuli from the EmpaToM paradigm (Kanske et al., 2015). We selected eight of the original sets of narrators, each presenting four narrations that varied in emotional valence (neutral vs. negative) and in the type of question they prompted (ToM Requirement: noToM vs. ToM). We then added two positively valenced narrations (one noToM, one ToM) and two narrations with embarrassing valence (one noToM, one ToM). Each set was recorded by a single amateur actor. For EmpaToM-pos (Pilot 1/Experiment 1), the neutral, negative, and positive narrations from each set were used. For EmpaToM-embarrass (Pilot 2/Experiment 2), the neutral, negative, and embarrassing narrations from each set were used. The following two example sets were translated into English from their original German versions by the authors. Correct answers to the corresponding questions are underlined.

S1.1 Example Sets of Narrations – German Version

Natalie

Geschichte 1 – Neutral & noToM

„Kaffee, nee vertrag ich nicht so gut... wegen dem Magen. Ich bin eher die Teetrinkerin. Zwei Liter Flüssigkeit am Tag- sollen ja ganz gesund sein. Manchmal gehe ich auch in den Wald und sammel Kräuter im Wald und mach dann so nen Mix daraus. Ist auch ganz gut zum Verschenken."

Es stimmt,

- <u>dass Natalie von Tee keine Magenschmerzen bekommt.</u>
- dass Natalie keinen industriell hergestellten Tee trinkt.
- dass Natalie nahe an einem Waldstück wohnt und dort Kräuter sammelt.

Geschichte 2 – Neutral & ToM

„Der Niklas hat jetzt mit Geige angefangen. Das hat er sich selbst ausgesucht... ich bin ganz schön stolz auf ihn. Zuerst hab ich mir ´n bisschen Sorgen gemacht wegen unserem Nachbarn, aber der hat's nicht so mit den Ohren. Der reagiert kaum, wenn man ihn grüßt."

Natalie denkt,

- <u>dass das Geigenspiel ihres Sohnes den Nachbarn nicht stört.</u>
- dass Geige ein gutes Instrument für Kinder ist.
- dass ihr Nachbar aus Unhöflichkeit nie zurückgrüßt.

Geschichte 3 – Negativ & noToM

„Der Druck ist eben so hoch, auf der Arbeit, alleine mit Kind... es ist alles so viel zu viel. Da muss ich mich einfach manchmal übergeben. Ich weiß überhaupt nicht, wie ich das schaffen soll...."

Es stimmt,

- <u>dass Natalie nicht mit dem Vater ihres Kindes zusammen wohnt.</u>
- dass Natalie eine Führungsposition inne hat.
- dass Natalie ein ungesundes Essverhalten hat.

Geschichte 4 – Negativ & ToM

„Ich hab mich so angestrengt für diese Festanstellung. Der Chef meinte, es wäre sicher... Und jetzt hat die Kollegin die Stelle bekommen und ich bin raus ab Ende Februar. Der hat einfach auf mehrere Pferde gesetzt…"

Natalie denkt,

- <u>dass Natalies Chef mehreren Kolleginnen Versprechungen gemacht hat.</u>
- dass sie gegenüber der Kollegin ein Vorrecht auf die Stelle hat.
- dass Natalies Chef wahrscheinlich mehrere Kolleginnen fest anstellen wird.

Taken from original EmpaToM; included in EmpaToM-pos and EmpaToM-embarrass

Geschichte 5 – Positiv & noToM

„Endlich hatten auch wir mal Glück! Ich habe eine neue Wohnung für Niklas und mich bekommen. Bezahlbar und viel größer als die andere. Jetzt bekommt Niklas auch endlich sein eigenes Zimmer, was er sich schon so lange gewünscht hat."

Es stimmt,

- <u>dass Natalie und ihr Sohn vorher auf eher engem Raum gewohnt haben.</u>
- dass Niklas sich vorher das Zimmer mit seinen Geschwistern teilen musste.
- dass die Miete für die neue Wohnung niedriger ist, als die alte war.

Geschichte 6 – Positiv & ToM

„Ich habe ja jetzt endlich nen top Babysitter für Niklas gefunden, da kann sogar ich kaum mithalten. Ja und deswegen war ich jetzt das erste Mal seit fünf Jahren wieder auf einem Date (*verlegenes, glückliches Grinsen*). Wir wollen uns jetzt öfter sehen."

Natalie denkt,

- <u>dass sie Niklas guten Gewissens mit dem Babysitter alleine lassen kann.</u>
- dass Niklas kein Problem damit haben wird, einen potentiellen Partner zu akzeptieren.
- dass sie sich das erste Mal seit langem wieder richtig amüsieren konnte.

Only included in EmpaToM-pos

Geschichte 7 – Peinlich & noToM

„Ich mache ja jeden Samstag einen Großeinkauf bei Rewe, dass ich unter der Woche nicht ständig in den Supermarkt springen muss. Ja, und an der Kasse, mega lange Schlange hinter mir, alle Artikel gescannt, fällt mir auf, dass ich meinen Geldbeutel vergessen hab."

Es stimmt,

- <u>dass der Supermarkt, in dem Natalie einkaufen war, gut besucht war.</u>
- dass Natalie mehrmals in der Woche in denselben Supermarkt einkaufen geht.
- dass Natalie ihr Smartphone als Alternative nutzen konnte, um zu bezahlen.

Geschichte 8 – Peinlich & ToM

„Als Niklas noch kleiner und so in einer richtigen Trotzphase war, hat er bei Wutanfällen in der Öffentlichkeit manchmal einfach so ‚Aua Aua' (Kind nachmachen) geschrien. Manche Blicke habe ich jetzt noch im Kopf…"

Natalie denkt,

- <u>dass die Passanten dachten, dass sie ihr Kind aus Überforderung schlägt.</u>
- dass Niklas Schmerzen nicht von Wut unterscheiden konnte.
- dass die Passanten sie für eine Rabenmutter halten, weil sie den Schmerz ihres Kindes nicht lindert.

Only included in EmpaToM-embarrass

Michael

Geschichte 1 – Neutral & noToM
„Ja, ich schreib' für ein Musik-Magazin... Wir machen da, wir machen da eigentlich alles Mögliche... aber insgesamt schon eher so Richtung Indie und so. Und… Ja, eigentlich höre ich den ganzen Tag Musik....”

Es stimmt,

- <u>dass das Magazin, bei dem Michael arbeitet, auch über andere Richtungen als Indie-Musik berichtet.</u>
- dass Michael selbst Indie-Musik produziert.
- dass das Magazin auch über andere Dinge als Musik berichtet.

Geschichte 2 – Neutral & ToM
„Ja, Jeff, mein Kollege, das ist echt ‘nen netter Kerl... aber manchmal da rastet der vollkommen aus. Wegen den kleinsten Kleinigkeiten! Und - wir wissen dann auch immer gar nicht, wie wir mit ihm umgehen sollen... Naja, ich mein, er entschuldigt sich dann auch immer - aber einfacher wird's dadurch auch nicht”

Michael denkt,

- <u>dass Jeff sich keine Mühe gibt, sein unangemessenes Verhalten zu verändern.</u>
- dass Jeff nicht weiß, dass sein Verhalten gegenüber den Kollegen unangemessen ist.
- dass Jeff gegen sein unangemessenes Verhalten nichts tun will.

Geschichte 3 – Negativ & noToM
„... jedenfalls als ich zurückkam war das ganze Haus in Flammen. Und die Versicherung hat auch nicht gezahlt... weil ich 'ne Kerze in meinem Zimmer hab brennen lassen. Ich hab' versucht meinen Eltern das Geld zurückzuzahlen, aber...”

Es stimmt,

- <u>dass Michael nicht zu Hause war, als das Feuer ausgebrochen ist.</u>
- dass das Haus von Michaels Eltern nicht versichert war.
- dass Michael und seine Eltern mit anderen Familien unter einem Dach wohnten.

Geschichte 4 – Negativ & ToM
„Eva hat gesagt sie würde die Pille nehmen... naja und dann – sie hat das Kind halt bekommen... und ich würde sie unterstützen, wirklich! Ich mein, ich zahl auch Unterhalt. Aber den Kleinen seh ich… seh ich praktisch nie.”

Michael denkt,

- <u>dass Eva ihn deutlich mehr Zeit mit seinem Sohn verbringen lassen soll.</u>
- dass seine Ex-Freundin ihn angelogen hat, damit sie von ihm schwanger werden konnte.
- dass Eva ihn für ihre Zwecke ausnutzt.

Taken from original EmpaToM; included in EmpaToM-pos and EmpaToM-embarrass

Geschichte 5 – Positiv & noToM
„Ich hab so lange auf dieses Lebensziel hingearbeitet, ich hab Trainingsplan gemacht, Intervalltraining, … und jetzt hab ich's endlich geschafft und bin meinen ersten Marathon gelaufen. Das ist einfach ein unbeschreibliches Gefühl, da über die Ziellinie zu laufen."

Es stimmt,

- <u>dass Michael sich gut in sportlichen Dingen auskennt.</u>
- dass Michael in der Vergangenheit an einem Marathon gescheitert ist.
- dass Michael einen professionell zusammengestellten Trainingsplan hatte.

Geschichte 6 – positiv & ToM
„Endlich keine Schmerzen mehr! Ich hatte jahrelang Rückenschmerzen, ich war bei tausenden Ärzten. Und immer wieder kam: "Ja, da kann man nichts machen" (*nachäffend*). Naja, und jetzt war ich bei diesem einen Physio, der hat mich *einmal* eingerenkt und seitdem geht's mir richtig gut. Ich bin wie ein neuer Mensch!"

Michael denkt,

- <u>dass die Ärzte, bei denen er war, keine Ahnung hatten, was sie tun.</u>
- dass Physiotherapeuten praktisch besser ausgebildet sind als Ärzte.
- dass er dank des Physiotherapeuten nie wieder Rückenschmerzen haben wird.

Only included in EmpaToM-pos

Geschichte 7 – peinlich & noToM
„Ich wollte halt diese eine WhatsApp noch zu Ende schreiben und bin dann völlig vertieft in mein Handy am Hauptbahnhof aus dem Bus ausgestiegen. Hm, naja, und dann hab ich halt dieses Schild nicht gesehen - wumms, voll davorgerannt. Vor all den Menschen."

Es stimmt,

- <u>dass Michael im Bus angefangen hat, eine Nachricht zu schreiben.</u>
- dass am Hauptbahnhof gerade nur wenige Menschen anwesend waren.
- dass Michael sonst eigentlich nicht beim Aussteigen auf sein Handy geschaut hat.

Geschichte 8 – peinlich & ToM
„Oh, ich hatte endlich mal wieder ein tolles Date – Julia heißt sie, genau so wie meine Schwester. Und ich wollte ihr dann einfach nochmal schreiben, wie wunderschön der Abend war. Und, naja, dann hab ich irgendwie die Chats verwechselt. Da kann ich mir bei der nächsten Familienfeier was anhören!"

Michael denkt,

- <u>dass seine Schwester ihn beim nächsten Familientreffen aufziehen wird.</u>
- dass seine Schwester beim nächsten Treffen alles über sein Date erfahren will.
- dass seine Schwester One-Night-Stands nicht gut findet und ihn verurteilt

.

Only included in EmpaToM-embarrass

S1.2 Example Sets of Narrations – English Translation

Natalie

Narration 1 – Neutral & noToM

"Coffee, no, I don't tolerate it very well... because of my stomach. I'm more of a tea drinker. Two liters of fluid a day—they say it's very healthy. Sometimes I go into the forest and collect herbs and then make a mixture out of them. It's also a nice gift."

It is true

- that Natalie doesn't get stomach ache from tea.
- that Natalie doesn't drink industrially produced tea.
- that Natalie lives near a forest and collects herbs there.

Narration 2 – Neutral & ToM

"Niklas has started playing the violin. He chose it himself... I'm really proud of him. At first I was a little worried about our neighbor, but he's not so good with his ears. He hardly reacts when you greet him."

Natalie thinks

- that her son's violin playing doesn't bother the neighbor.
- that the violin is a good instrument for children.
- that her neighbor never says hello back out of rudeness.

Narration 3 – Negative & noToM

"The pressure is just so high, at work, alone with a child... it's all too much. Sometimes I just have to throw up. I have no idea how I'm going to manage..."

It is true

- that Natalie does not live with the father of her child.
- that Natalie holds a management position.
- that Natalie has unhealthy eating habits.

Narration 4 – Negative & ToM

"I worked so hard for this permanent position. The boss said it was a sure thing... And now my colleague got the job and I'm out at the end of February. He just hedged his bets..."

Natalie thinks

- that Natalie's boss made promises to several colleagues.
- that she has priority over her colleague for the job.
    - that Natalie's boss will probably hire several colleagues permanently.

Taken from original EmpaToM; included in EmpaToM-pos and EmpaToM-embarrass

Narration 5 – Positive & noToM

"Finally, we got lucky! I found a new apartment for Niklas and me. It's affordable and much bigger than the other one. Now Niklas will finally get his own room, which he's wanted for so long."

It is true

- that Natalie and her son previously lived in rather cramped quarters.
- that Niklas previously had to share a room with his siblings.
- that the rent for the new apartment is lower than the old one.

Narration 6 – Positive & ToM

"I've finally found a great babysitter for Niklas - even I can hardly keep up. Yeah, and that's why I went on a date for the first time in five years (*embarrassed, happy grin*). We want to see each other more often now."

Natalie thinks

- that she can leave Niklas alone with the babysitter with a clear conscience.
- that Niklas will have no problem accepting a potential partner.
- that she was able to really enjoy herself for the first time in a long time.

Only included in EmpaToM-pos

Narration 7 – Embarrassing & noToM

"I do a big shop at Rewe [German supermarket] every Saturday so that I don't have to keep popping to the supermarket during the week. So, at the checkout, with a really long line behind me and all my items scanned, I realize that I've forgotten my wallet."

It is true

- that the supermarket where Natalie was shopping was busy.
- that Natalie goes shopping at the same supermarket several times a week.
- that Natalie was able to use her smartphone as an alternative to pay.

Narration 8 – Embarrassing & ToM

"When Niklas was younger and going through a real defiant phase, he would sometimes just scream 'Ouch, ouch' (imitating child) during tantrums in public. I can still remember some of the looks I got..."

Natalie thinks

- that passers-by thought she was hitting her child because she was overwhelmed.
- that Niklas couldn't distinguish pain from anger.
- that passers-by thought she was a bad mother because she wasn't alleviating her child's pain.

Only included in EmpaToM-embarrass

Michael

Narration 1 – Neutral & noToM

"Yeah, I write for a music magazine... We actually cover all kinds of things... but overall it's more in the indie direction. And… yeah, basically I listen to music all day...."

It is true

- that the magazine Michael works for also reports on genres other than indie music.
- that Michael himself produces indie music.
- that the magazine also reports on things other than music.

Narration 2 – Neutral & ToM

"Yeah, Jeff, my colleague, he's really a nice guy... but sometimes he completely loses it.. Over the smallest little things! And – we never really know how to deal with him then... Well, I mean, he always apologizes afterwards – but that doesn't really make it easier."

Michael thinks

- that Jeff doesn't make any effort to change his inappropriate behavior.
- that Jeff doesn't know his behavior toward his colleagues is inappropriate.
- that Jeff doesn't want to do anything about his inappropriate behavior.

Narration 3 – Negative & noToM

"... anyway, when I came back the whole house was in flames. And the insurance didn't pay... because I had left a candle burning in my room. I tried to pay my parents back the money, but..."

It is true

- that Michael wasn't at home when the fire broke out.
- that Michael's parents' house was not insured.
- that Michael and his parents lived under one roof with other families.

Narration 4 – Negative & ToM

"Eva said she was taking the pill... well and then – she just had the baby... and I would have supported her, really! I mean, I do pay child support. But I practically never see the little one."

Michael thinks

- that Eva should let him spend much more time with his son.
- that his ex-girlfriend lied to him so she could get pregnant by him.
- that Eva is exploiting him for her own purposes.

Taken from original EmpaToM; included in EmpaToM-pos and EmpaToM-embarrass

Narration 5 – Positive & noToM

"I worked toward this goal for so long, I made a training plan, interval training... and now I've finally done it and run my first marathon. It's just an indescribable feeling to cross the finish line."

It is true

- that Michael knows a lot about sports.
- that Michael failed to complete a marathon in the past.
- that Michael had a professionally designed training plan

Narration 6 – Positive & ToM

"No more pain at last! I had back pain for years, I went to thousands of doctors. And all I kept hearing was: 'Yeah, nothing can be done' (*mocking*). Well, and then I went to this one physiotherapist, he adjusted me once and since then I feel really good. I'm like a new person!"

Michael thinks

- that the doctors he went to had no idea what they were doing.
- that physiotherapists are practically better trained than doctors.
- that thanks to the physiotherapist he will never have back pain again.

Only included in EmpaToM-pos

Narration 7 – Embarrassing & noToM

"I just wanted to finish that one WhatsApp message and then got completely absorbed in my phone when I got off the bus at the main station. Hm, well, and then I just didn't see the sign – wham, walked straight into it. In front of all those people."

It is true

- that Michael started writing a message on the bus.
- that only a few people were present at the main station at the time.
- that Michael usually didn't look at his phone while getting off the bus.

Narration 8 – Embarrassing & ToM

"Oh, I finally had a great date again – her name's Julia, just like my sister. And then I just wanted to text her again to say how wonderful the evening was. And, well, I somehow mixed up the chats. Now I'll get an earful at the next family gathering!"

Michael thinks

- that his sister will tease him at the next family gathering.
- that his sister will want to hear all about his date at the next meeting.
- that his sister doesn't approve of one-night stands and will judge him.

Only included in EmpaToM-embarrass

S2 Empathy Induction in Individual Sets

In addition to the main analyses, we examined affect ratings separately for each narrator set (i.e., the eight videos provided by each speaker). To assess whether positive empathy was successfully elicited, we compared affect ratings between neutral and positive trials within each set of Pilot 1/Experiment 1. To assess whether empathic embarrassment was successfully elicited, we compared embarrassment ratings between neutral and embarrassing trials within each set of Pilot 2/Experiment 2. Across both the pilot studies and the higher-powered experiments, these differences in ratings were significant for all narrator sets. After the pilot, we re-recorded two narration sets with new actors to improve stimulus quality: ‘Klara’, which did not sufficiently elicit positive affect, and ‘Tobias’, in which neutral and positive trials were rated too similarly.

Pilot 1: Affect Rating – neutral vs. positive trials

Set	$M_{neutral}$	$M_{positive}$	$SE_{neutral}$	$SE_{positive}$	*t*	*df*	*p*	*d*	
Hannelore	0.62	1.20	0.12	0.14	-5.00	27	<.001	-0.95	
Hartmut	0.59	1.41	0.13	0.17	-6.01	27	<.001	-1.14	
Ivko	0.11	0.77	0.12	0.18	-4.77	27	<.001	-0.90	
Klara	-0.20	0.66	0.10	0.16	-4.92	27	<.001	-0.93	*
Michael	-0.12	1.04	0.09	0.15	-6.51	27	<.001	-1.23	
Natalie	0.30	1.25	0.11	0.17	-6.68	27	<.001	-1.26	
Sabine	-0.05	0.82	0.06	0.15	-4.98	27	<.001	-0.94	
Tobias	0.55	0.93	0.11	0.13	-3.29	27	.003	-0.62	*

*: Sets selected for revision after pilot. Klara: Insufficient elicitation of positive affect; Tobias: Insufficient distinction between neutral and negative narrations

Pilot 2: Embarrassment Rating – neutral vs. embarrassing trials

Set	$M_{neutral}$	M_{embarr}	$SE_{neutral}$	SE_{embarr}	*t*	*df*	*p*	*d*
Hannelore	0.87	2.02	0.17	0.30	-5.11	26	<.001	-0.98
Hartmut	0.28	1.70	0.14	0.28	-6.45	26	<.001	-1.24
Ivko	0.46	2.37	0.16	0.27	-7.80	26	<.001	-1.50
Klara	0.57	2.56	0.16	0.33	-7.98	26	<.001	-1.54
Michael	0.39	2.35	0.11	0.31	-7.47	26	<.001	-1.44
Natalie	0.39	1.98	0.17	0.31	-5.23	26	<.001	-1.01
Sabine	0.56	1.94	0.16	0.28	-6.12	26	<.001	-1.18
Tobias	0.80	2.48	0.23	0.33	-6.02	26	<.001	-1.16

Experiment 1: Affect Rating – neutral vs. positive trials

Set	$M_{neutral}$	$M_{positive}$	$SE_{neutral}$	$SE_{positive}$	t	df	p	d
Hannelore	0.64	1.10	0.11	0.12	-3.85	59	<.001	-0.50
Hartmut	0.34	1.14	0.08	0.11	-9.41	59	<.001	-1.21
Ivko	0.12	0.68	0.08	0.12	-5.19	59	<.001	-0.67
Klara*	-0.15	0.92	0.08	0.12	-9.10	59	<.001	-1.17
Michael	-0.02	1.15	0.07	0.15	-7.36	59	<.001	-0.95
Natalie	0.25	1.05	0.09	0.12	-8.90	59	<.001	-1.15
Sabine	0.05	0.83	0.07	0.10	-8.35	59	<.001	-1.08
Tobias*	0.03	0.78	0.09	0.13	-7.18	59	<.001	-0.93

*: Sets re-recorded after pilot

Experiment 2: Embarrassment Rating – neutral vs. embarrassing trials

Set	$M_{neutral}$	M_{embarr}	$SE_{neutral}$	SE_{embarr}	t	df	p	d
Hannelore	0.78	1.81	0.12	0.17	-5.74	54	<.001	-0.77
Hartmut	0.22	1.71	0.08	0.17	-8.75	54	<.001	-1.18
Ivko	0.48	2.25	0.11	0.20	-10.27	54	<.001	-1.39
Klara*	0.53	2.16	0.10	0.19	-8.57	54	<.001	-1.16
Michael	0.55	2.00	0.13	0.17	-7.90	54	<.001	-1.07
Natalie	0.51	1.83	0.12	0.18	-6.55	54	<.001	-0.88
Sabine	0.56	1.77	0.12	0.16	-6.97	54	<.001	-0.94
Tobias*	0.87	2.11	0.15	0.20	-4.75	54	<.001	-0.64

*: Sets re-recorded after pilot

S3 Question Accuracy in Single-Choice Questions Per Narration

We assessed the accuracy of the single-choice questions targeting comprehension of the narrations to ensure comparable difficulty across conditions, both between valence conditions (EmpaToM-pos: neutral, negative, positive; EmpaToM-embarrass: neutral, negative, embarrassing) and, importantly, between questions requiring Theory of Mind (ToM) versus purely factual reasoning (noToM).

Fifteen questions were flagged in the pilots for extreme accuracy levels (four very high, 11 very low), indicating they may not reliably capture meaningful differences in responses. These questions were revised for Experiments 1 and 2. The revisions led to slightly lower accuracy in the newly added valence conditions (positive/embarrassing). The tables below highlight specific questions that future researchers might consider revising further if their study requires equivalent difficulty across valence conditions.

Conditions

1: neutral – noToM
2: neutral – ToM
3: negative – noToM
4: negative – ToM
5: positive – noToM
6: positive – ToM
7: embarrassing – noToM
8: embarrassing - ToM

Conditions
1: neutral – noToM
2: neutral – ToM
3: negative – noToM
4: negative – ToM
5: positive – noToM
6: positive – ToM

Question accuracy – EmpaToM-pos

Video	Pilot 1	Exp. 1
Hannelore1	0.57	0.67*
Hannelore2	0.75	0.90
Hannelore3	0.46	0.92*
Hannelore4	0.64	0.65
Hannelore5	0.68	0.72
Hannelore6	0.93	0.73
Hartmut1	0.75	0.83
Hartmut2	0.75	0.88
Hartmut3	0.89	0.93
Hartmut4	0.93	0.90
Hartmut5	0.71	0.67
Hartmut6	0.93	0.93
Ivko1	0.75	0.82
Ivko2	0.43	0.65*
Ivko3	0.86	0.77
Ivko4	0.89	0.87
Ivko5	0.89	0.87
Ivko6	0.64	0.68
Klara1	0.79	0.80
Klara2	0.39	0.75*
Klara3	0.50	0.60
Klara4	1.00	0.70*
Klara5	0.57	0.63
Klara6	0.93	0.87
Michael1	0.46	0.72*
Michael2	0.79	0.80
Michael3	0.86	0.88
Michael4	0.57	0.55
Michael5	1.00	0.27*[a]
Michael6	0.11	0.40*
Natalie1	0.82	0.80
Natalie2	0.86	0.80
Natalie3	0.43	0.67*
Natalie4	0.86	0.88
Natalie5	0.89	0.82
Natalie6	0.79	0.48
Sabine1	0.79	0.80
Sabine2	0.57	0.72*
Sabine3	0.64	0.73*
Sabine4	0.79	0.72
Sabine5	0.89	0.90
Sabine6	0.86	0.85
Tobias1	0.54	0.75
Tobias2	0.86	0.82
Tobias3	0.93	0.83
Tobias4	0.46	0.68*
Tobias5	0.50	0.58[a]
Tobias6	1.00	0.55*[a]

*: revised after pilot. [a]: potential for revision in future studies

Conditions
1: neutral – noToM
2: neutral – ToM
3: negative – noToM
4: negative – ToM
7: embarrassing – noToM
8: embarrassing – ToM

Question accuracy – EmpaToM-embarrass

Video	Pilot 1	Exp. 1
Hannelore1	0.44	0.60*
Hannelore2	0.74	0.80
Hannelore3	0.59	0.87*
Hannelore4	0.74	0.58
Hannelore7	0.67	0.67
Hannelore8	0.93	0.85
Hartmut1	0.70	0.78
Hartmut2	0.89	0.82
Hartmut3	0.85	0.84
Hartmut4	0.89	0.87
Hartmut7	0.56	0.53
Hartmut8	0.74	0.78
Ivko1	0.63	0.73
Ivko2	0.59	0.49*
Ivko3	0.70	0.76
Ivko4	0.70	0.78
Ivko7	0.56	0.53
Ivko8	0.74	0.49
Klara1	0.85	0.91
Klara2	0.33	0.78*
Klara3	0.70	0.65
Klara4	0.93	0.65*
Klara7	0.96	0.65*
Klara8	0.70	0.40[a]
Michael1	0.41	0.69*
Michael2	0.59	0.75
Michael3	0.85	0.93
Michael4	0.63	0.45
Michael7	0.89	0.78
Michael8	0.85	0.95
Natalie1	0.78	0.75
Natalie2	0.74	0.80
Natalie3	0.41	0.58*
Natalie4	0.85	0.95
Natalie7	0.93	0.87
Natalie8	0.48	0.35*[a]
Sabine1	0.74	0.73
Sabine2	0.33	0.71*
Sabine3	0.48	0.82*
Sabine4	0.56	0.71
Sabine7	0.74	0.62
Sabine8	0.93	0.93
Tobias1	0.67	0.65
Tobias2	0.74	0.80
Tobias3	0.89	0.80
Tobias4	0.52	0.73*
Tobias7	0.56	0.47[a]
Tobias8	0.70	0.55

*: revised after pilot. [a]: potential for revision in future studies

S4 Inter-Individual Correlations

We examined interindividual associations among key dependent variables using Pearson correlations. Negative empathic responding (affect in negative vs. neutral trials) was correlated with ToM accuracy, overall prosociality, and prosociality in negative trials. Positive empathy (affect in positive vs. neutral trials; Pilot 1/Exp. 1) and empathic embarrassment (embarrassment in embarrassing vs. neutral trials; Pilot 2/Exp. 2) were examined similarly, including associations with ToM performance, overall prosociality, and prosociality specifically in positive or embarrassing trials, respectively. In addition, correlations between negative empathic responding and positive empathy (Pilot 1/Exp. 1) or empathic embarrassment (Pilot 2/Exp. 2) were computed. Finally, we examined correlations between ToM accuracy and overall prosociality for each experiment. The following tables report Pearson's r values and significance indicators (*: $p < .05$, **: $p < .01$, ***: $p < .001$)

**Pilot 1: Correlations between Dependent Variables**		
Empathy negative trials	ToM accuracy	*r* = .03
	Prosociality overall	*r* = .66***
	Prosociality negative trials	*r* = .71***
Empathy positive trials	ToM accuracy	*r* = -.02
	Prosociality overall	*r* = .70***
	Prosociality positive trials	*r* = .66***
Empathy negative trials	Empathy positive trials	*r* = .75***
ToM accuracy	Prosociality overall	*r* = .00

**Pilot 2: Correlations between Dependent Variables**		
Empathy negative trials	ToM accuracy	*r* = .35
	Prosociality overall	*r* = .46*
	Prosociality negative trials	*r* = .60***
Empathy embarrassing trials	ToM accuracy	*r* = .21
	Prosociality overall	*r* = .22
	Prosociality embarrassing trials	*r* = .25
Empathy negative trials	Empathy embarrassing trials	*r* = .56**
ToM accuracy	Prosociality overall	*r* = .02

**Experiment 1: Correlations between Dependent Variables**		
Empathy negative trials	ToM accuracy	*r* = .04
	Prosociality overall	*r* = .36**
	Prosociality negative trials	*r* = .54***
Empathy positive trials	ToM accuracy	*r* = -.05
	Prosociality overall	*r* = .47***
	Prosociality positive trials	*r* = .40**
Empathy negative trials	Empathy positive trials	*r* = .74***
ToM accuracy	Prosociality overall	*r* = .15

**Experiment 2: Correlations between Dependent Variables**		
Empathy negative trials	ToM accuracy	*r* = .23
	Prosociality overall	*r* = .22
	Prosociality negative trials	*r* = .21
Empathy embarrassing trials	ToM accuracy	*r* = .27*
	Prosociality overall	*r* = .01
	Prosociality embarrassing trials	*r* = .01
Empathy negative trials	Empathy embarrassing trials	*r* = .56**
ToM accuracy	Prosociality overall	*r* = -.06

S5 Suggested Set Selection for Balanced Difficulty

The task evaluation showed that question accuracy (ToM/factual reasoning) was lower in the newly added positive and embarrassing conditions, which may be problematic for use cases in which the valence conditions should not differ in baseline difficulty. To make the task easier to adapt for future use, we provide the full question texts and response options.

Below, we additionally suggest sets of narrations that are balanced in terms of accuracy (based on the data from the task evaluation study), meaning that they do not differ significantly across emotional valence. To achieve this, the original eight narrators were reduced to six. For the EmpaToM-pos, the narrations by “Tobias” and “Michael” were excluded. For the EmpaToM-embarrass, it was not possible to remove two complete narrator sets; instead, narrations by “Tobias” were excluded, and narrations from “Hartmut” and “Ivko” were combined.

The suggested sets are presented in the table below.

EmpaToM-pos	EmpaToM-embarrass
Hannelore1	Hannelore1
Hannelore2	Hannelore2
Hannelore3	Hannelore3
Hannelore4	Hannelore4
Hannelore5	Hannelore7
Hannelore6	Hannelore8
Hartmut1	-
Hartmut2	-
Hartmut3	-
Hartmut4	-
Hartmut5	Hartmut7
Hartmut6	Hartmut8
Ivko1	Ivko1
Ivko2	Ivko2
Ivko3	Ivko3
Ivko4	Ivko4
Ivko5	-
Ivko6	-
Klara1	Klara1
Klara2	Klara2
Klara3	Klara3
Klara4	Klara4
Klara5	Klara7
Klara6	Klara8
-	Michael1
-	Michael2
-	Michael3
-	Michael4
-	Michael7
-	Michael8
Natalie1	Natalie1
Natalie2	Natalie2
Natalie3	Natalie3
Natalie4	Natalie4
Natalie5	Natalie7
Natalie6	Natalie8
Sabine1	Sabine1
Sabine2	Sabine2
Sabine3	Sabine3
Sabine4	Sabine4
Sabine5	Sabine7
Sabine6	Sabine8
-	-
-	-
-	-
-	-
-	-
-	-
